# Identification of Target PTEN-Based miR-425 and miR-576 as Potential Diagnostic and Immunotherapeutic Biomarkers of Colorectal Cancer With Liver Metastasis

**DOI:** 10.3389/fonc.2021.657984

**Published:** 2021-08-19

**Authors:** Xiaoyun Hu, Qiuchen Chen, Hao Guo, Kuo Li, Boshi Fu, Yu Chen, Haishan Zhao, Minjie Wei, Yalun Li, Huizhe Wu

**Affiliations:** ^1^Department of Pharmacology, School of Pharmacy, China Medical University, Shenyang, China; ^2^Liaoning Key Laboratory of Molecular Targeted Anti-tumor Drug Development and Evaluation, Liaoning Cancer Immune Peptide Drug Engineering Technology Research Center, Key Laboratory of Precision Diagnosis and Treatment of Gastrointestinal Tumors, Ministry of Education, China Medical University, Shenyang, China; ^3^Department of Gene Detection, Liaoning Medical Diagnosis and Treatment Center, Shenyang, China; ^4^Department of Anorectal Surgery, First Hospital of China Medical University, Shenyang, China

**Keywords:** miR-425, miR-576, PTEN, biomarker, immunotherapy, colorectal cancer liver metastasis

## Abstract

A major complication of colorectal cancer (CRC), one of the most common and fatal types of cancers, is secondary liver metastasis. For patients with this fate, there are very few biomarkers available in clinical application, and the disease remains incurable. Recently, increasing studies demonstrated that tumorigenesis and development are closely related to immune escape, indicating that the roles of immune-related indicators might have been neglected in the past in colorectal cancer liver metastases (CRLM). Here, we unveil that elevated miR-425 and miR-576 promote CRLM through inhibiting PTEN-mediated cellular immune function. Specifically, miR-425 and miR-576 were identified for their significant upregulation in CRLM compared with the primary CRC tissues based on GSE81581 (*n* = 8) and GSE44121 (*n* = 18) datasets. Besides, we determined that the two microRNAs (miRNAs) coparticipated in restraining P53 and transforming growth factor beta (TGF-β) signaling pathways associated with tumor metastasis, and both shortened the overall survival of the patients with metastatic susceptibility. Notably, *in situ* hybridization on relatively large samples of paired CRC tissues (*n* = 157) not only substantiated that the expression of miR-425 and miR-576 was dramatically upregulated in CRLM but also revealed that they were closely related to tumor deterioration, especially liver metastases. Moreover, we further confirmed that the combination of miR-425 and miR-576 was an effective predictive model for liver metastases and poor clinical outcomes. Mechanically, downregulated PTEN (GSE81558, *n* = 6) was verified to be a shared target of miR-425 and miR-576 acting as metastasis-related oncogenes, on account of the presence of binding sites (+2928–+2934 and +4371–+4378, respectively) and the collaborative suppression of P53/TGF-β signaling in CRLM, which was further confirmed in CRC cells (HCT116 and SW480) based on systematic molecular biology experiments. Importantly, the target *PTEN* was strongly associated with microsatellite instability, tumor microenvironment, and immune cell infiltration. Thus, we speculate that miR-425 and miR-576 are novel biomarkers for CRLM prevention and immunotherapy and upstream inhibitors of the PTEN-P53/TGF-β function axis.

## Introduction

Colorectal cancer (CRC) is the third most common cancer in the western world and represents a leading cause of death worldwide (1). More than half of CRC patients with a low tumor–node–metastasis (TNM) stage at diagnosis usually develop metastases located in the liver within 3 years (2), indicating the urgent need for efficient biomarkers to assess the susceptibility. In addition, although surgical resection could provide these patients with an increase in 5-year survival, the heterogeneity of colorectal cancer liver metastases (CRLMs) leads to the fact that prediction and improvement of individual outcomes after surgery remain a challenge (3, 4). Recently, there is no lack of exploration of targets especially miRNAs for CRLM (5), but few ones could be applied in clinical practice, including immunotherapy, an emerging therapeutic strategy for cancer. Therefore, one of the primary research focuses is still to excavate potential biomarkers and immunotherapeutic targets for both early evaluation and accurate treatment of CRLM.

After their discovery, microRNAs (miRNAs) have been revealed to play crucial roles in cancer biology (6). Of note, due to the high structure stability and tissue specificity of these short non-coding RNA (20–25 nucleotides), increasing evidence highlights their capacity as a new class of valuable biomarkers (7, 8). Current research suggests that the impact of miRNAs in CRLM also catches much attention, and a few miRNAs, such as miR-122, miR-200c, and miR-338, have been proven to be promising in predicting such tumorigenesis or prognosis (9–11). However, the limitation of sample size and the insensitivity of single-miRNA signature greatly hinder the further related exploration. hsa-miR-425-5p (miR-425) and hsa-miR-576-3p (miR-576) in our study are mature and functional, which makes them as potential biomarkers for occurrence, development, or prognosis of tumors such as liver cancer, gastric cancer, and bladder cancer (12–14). At present, the exploration of the clinical values of the two miRNAs still stays in primary CRC (15, 16), while almost no liver metastasis is involved. On the other hand, the anti-miRNA treatment is also cutting a figure in cancer treatment, and the delivery of anti-miRNAs has been implemented for triple-negative breast cancer (TNBC), chondrosarcoma, and cholangiocarcinoma (CCA) therapy (17–19). However, for immunotherapy in cancers especially in CRLM, no miRNA-based strategy has been reported yet, suggesting that more potential targets should be explored for promoting further studies in this field.

It is widely known that miRNAs usually play biological roles by regulating gene expression at the posttranscriptional level through interacting with 3′-untranslated regions (3′UTRs) of the target messenger RNAs (mRNAs) (20). Therefore, the function of the target genes determines the predictive and therapeutic potential of miRNAs (21–24). Phosphatase and tensin homologue deleted on chromosome 10 (PTEN), a tumor suppressor, is closely associated with malignant progression in many cancers, including CRLM (25). Naturally, PTEN was identified as the specific target of a few miRNAs such as miR-181a and miR-298 involved in controlling CRLM (26, 27). Of note, a recent study revealed that miR-25-3p, miR-130b-3p, and miR-425-5p jointly targeted PTEN, thereby promoting the CRLM occurrence (28). However, there are very few focuses on the regulatory relationship between miR-576-3p and PTEN. Moreover, PTEN was widely received to play the pivotal function through negatively regulating the PI3K/Akt signaling pathway (29, 30), while it is necessary to look for new downstream regulatory mechanisms. Importantly, several studies demonstrate the new lights on the roles of PTEN in immunotherapy of various types of cancers except for CRLM (31–33), which hints us to fill the gap of PTEN-mediated immunoregulation in such tumor.

Herein, we first identified miR-425 and miR-576 for their significant upregulation and participation in P53/transforming growth factor beta (TGF-β) signaling pathways in CRLM. Thereafter, we performed *in situ* hybridization (ISH) assay on the larger sample cohort to further verify the dysregulation of the two indicators in CRC (*n* = 157), especially that in CRLM (*n* = 29). Besides, we conducted a series of analyses to confirm that the two-miRNA signature was closely related to liver metastasis and clinical outcomes based on these CRC patients. Furthermore, PTEN was ascertained as the direct target of miR-425 and miR-576 cooperating in the immune escape-dependent tumor progression in CRLM. In brief, on the basis of revealing the miR-425/miR-576-PTEN-P53/TGF-β signaling axis, we defined miR-425 and miR-576 as potential biomarkers and immunotherapeutic targets for CRLM prevention and treatment.

## Materials and Methods

### Collection of CRC Patient Specimens

In our study, we recruited 157 paired CRC tissues and matched adjacent-tumor controls based on the First Hospital of China Medical University and Cancer Hospital of China Medical University from September 2014 to September 2015. All enrolled patients were diagnosed histopathologically with clear colorectal cancer, 29 of which had secondary liver metastases. Clinically, enhanced CT, enhanced MR, and standard reviews of serum tumor biomarkers were used to detect the progression of CRLM. We excluded those patients with a history of other malignant tumors or without receiving chemotherapy or radiation before surgery. All patients signed the written informed consent form before being enrolled in this study. The Medical Ethics Committee of China Medical University approved this study. The collected specimens were stored at −80°C before being used. The clinicopathological variables of these included CRC patients were integrally collected, among whom the follow-up end points involved were CRC progression or patient death, and the follow-up time was 110 months. All the relevant details are presented in **Supplementary Table S1**.

### Data From GEO and TCGA

RNA-seq data were collected from Gene Expression Omnibus (GEO) and The Cancer Genome Atlas (TCGA). Among them, the raw data of the primary CRC tissues and paired liver metastases [GSE81581 (*n* = 8), GSE44121 (*n* = 18), GSE81558 (*n* = 6), and GSE62321 (*n* = 26)] were downloaded from GEO database (https://www.ncbi.nlm.nih.gov/gds/). Moreover, TCGA data used for further analyses were downloaded from the TCGA website (https://www.cancer.gov/about-nci/organization/ccg/research/structural-genomics/tcga) or obtained from the online platforms.

### Gene Expression Analysis

Differentially expressed genes (DEGs), with statistical significance between the primary CRC tissues and liver metastases, were identified by running Limma R package and shown as volcano plot (Biomarker Technologies, http://www.biomarker.com.cn/), and the threshold values of up- and downregulated DEGs were set at |Log_2_ FC| > 1 and *p* ≤ 0.05. Besides, hierarchical clustering was performed to reveal the relationships of the DEGs in our included samples based on pheatmap R package. To reflect the filtering process, we produced a Venn diagram *via* Venn R package to show all possible mRNAs among a finite collection of the sets that contained the downregulated DEGs and the targets of both miR-425 and miR-576 based on the data in GSE81558 (*n* = 6) and the intersectional mRNAs of the three sets were used for further analysis.

### Function Enrichment Analysis

The signaling pathways miR-425 and miR-576 involved were enriched by Kyoto Encyclopedia of Genes and Genomes (KEGG) analysis based on the DIANA TOOL-mirPath v.3 database (http://snf-515788.vm.okeanos.grnet.gr/), and the visualization of data was carried out through the Enrichment Analysis Circle Diagram Tool of SangerBox platform (http://sangerbox.com/). Specifically, we inputted four columns of data, which are term, number of genes, *p* value, and name of genes, then selected the key colors and drew the ostensive images. Moreover, gene set enrichment analysis (GSEA) was used to investigate the mechanisms related to *PTEN* high/low expression in CRLM. We considered that the statistically significant differences were set at normalized enrichment score (NES) >1, nominal (NOM) *p* ≤ 0.05, and false discovery rate (FDR) <25%.

### Molecular Interaction Prediction

The downstream targets of miR-425 and miR-576 binding to mRNA 3′UTR were predicted by TargetScan database (http://www.targetscan.org/vert_72/). We entered a human gene symbol (PTEN) in the specific box and just submitted the request. Protein–protein interaction networks analysis was performed by STRING (http://string-db.org/cgi/input.pl) to identify the factors interacting directly with the selected protein. In our study, we searched PTEN *via Homo sapiens* module, and the top 10 proteins were obtained. Besides, RNAhybrid platform (https://bibiserv.cebitec.uni-bielefeld.de/rnahybrid) was used to evaluate the binding site and minimum free energy (MFE) of miRNA (miR-425/miR-576)–mRNA (*PTEN*) interaction. In brief, we entered the FASTA sequences of the two miRNAs and PTEN mRNA in the Submission module, checked the Generate graphics options, and started the calculations of interaction between these RNAs.

### TMA and ISH Assays

Tissue microarrays (TMAs) were performed according to a previous publication with some modifications (34). Briefly, each 4-μm TMA section was deparaffinized and rehydrated in a graded ethanol series. Furthermore, for RNA *in situ* hybridization (ISH) assay, we determined miR-425 and miR-576 levels according to the manufacturer’s protocol (Boster, Wuhan, China). Briefly, the TMA section was incubated with digoxin-labeled probes solutions overnight at 37°C. Then, it was exposed in the streptavidin–peroxidase reaction system and stained with 3,3′-diaminobenzidine (DAB). Finally, the expression of miR-425 and miR-576 was counted under a microscope (Nikon, Tokyo, Japan) for subsequent analysis. In our study, expression scores = (negative percentage × 0) + (weak positive percentage × 1) + (medium positive percentage × 2) + (strong positive percentage × 3); and the expression scores ranged from 0 to 300. The specific sequences of the probe are listed in **Supplementary Table S2**.

### Risk Score

We calculated risk scores of the prognostic model based on miR-425 and miR-576 expression (0–300) in CRC *via* multivariate Cox regression. As a result, risk score = 0.4253 * miR-425 + 0.4648 * miR-576, and the risk scores of each patient were shown in the continuous bar charts. Then, patients were divided into high- or low-risk groups according to the median for further survival analysis, and the prognostic assessment efficacy of two-factor was shown as the ROC (receiver operating character) and Kaplan–Meier curves.

### Immune Analysis

The relationships of miR-425/miR-576 and *PTEN* with immune checkpoints, immune pathways, microsatellite instability (MSI), tumor microenvironment (TME), and immune cell infiltration in CRLM were analyzed and visualized by Sangerbox Online platform (http://sangerbox.com/). Concretely, we used Pearson correlation analysis to reveal the associations between these indicators and immune checkpoints, immune pathways, and MSI. Importantly, TME evaluation including ESTIMATE Score, Immune Score (B cell, CD4 T cell, CD8 T cell, neutrophil, macrophage, and dendritic cell) (35), and Stromal score [cancer-associated fibroblasts (CAFs), myofibroblasts, myeloid cells, endothelial cells, and mesenchymal stromal cells (MSCs)] (36), used to characterize the tumor purity, was calculated based on the ESTIMATE algorithm (37). Besides, the abundances of immune infiltrates were evaluated by multiple immune deconvolution methods (38).

### Cell Culture

The human CRC cells (HCT116 and SW480) and HEK293T were purchased from Peking Union Medical College Cell Resource Center (PUMCCRC, Beijing, China). HCT116 and HEK293T cells were cultured in Dulbecco’s modified Eagle’s medium (DMEM) medium (Invitrogen, NY, USA), and SW480 cells were grown in L15 medium (HyClone, Logan, UT, USA) supplemented with 10% (*v/v*) fetal bovine serum (FBS, Gibco, NY, USA). These cells were maintained at 37°C with 100 U/ml penicillin/streptomycin (Invitrogen, CA, USA) in a humidified atmosphere with 5% CO_2_. In this study, an absence of mycoplasma or bacterial contamination of the cells was detected.

### Cell Transfection

The mimics and inhibitors of hsa-miR-425 and hsa-miR-576 were obtained from Ribobio (Guangzhou, China). Cells were cultured on six-well plates with a density of 60%–70%, then transiently transfected using Lipofectamine 2000 (Invitrogen, USA). Cells were collected after transfection at 24 or 48 h for further experiments.

### qPCR Assays

Briefly, the total RNAs were extracted by TRIzol reagent (Invitrogen, CA, USA), and the Reverse Transcription Kit was purchased from Vazyme (Nanjing, China). Besides, quantitative PCR (qPCR) was determined using ChamQ SYBR qPCR Master Mix (Low ROX Premixed) (Vazyme, Nanjing, China) in triplicate. The normalized relative expression of the indicator was analyzed using 2^−ΔΔCt^ method to the reference gene GAPDH. The primers of *PTEN* mRNA used for qPCR amplification were 5′-TAGAGCGTGCAGATAATGAC-3′ (forward) and 5′-GGCTCCTCTACTGTTTTTGT-3′ (reverse).

### Western Blot Assay

In brief, an equal amount of protein was separated with 10%–12% sodium dodecyl sulfate polyacrylamide gel (SDS-PAGE) and transferred to 0.22-μm polyvinylidene fluoride (PVDF) membranes (Millipore, Bedford, MA, USA). Thereafter, the membranes were blocked with 5% milk and immunoblotted with primary antibodies. The antibodies include PTEN (1:1,000; Bioss, Beijing, China), P53 (1:500; Bioss, Beijing, China), TGF-β (1:1000; Bioss, Beijing, China), and GAPDH (1:2000; Zsbio, Beijing, China).

### Luciferase Reporter Assay

HEK293T and HCT116 cells were seeded into 96-well plates overnight before transfection. Then, cells were cotransfected with luciferase reporter vectors [PTEN 3′UTR-WT and PTEN 3′UTR-Mut (for miR-425 and miR-576)] (100 ng/well) and miR-425 and/or miR-576 mimics (50 nM/well) by Lipofectamine 2000 (Invitrogen, CA, USA). Forty-eight hours posttransfection, the cells were collected and lysed in passive lysis buffer (Promega, WI, USA). The luciferase activities of the cell lysates were determined in a Nano-Glo^®^ Reporter Assay System.

### Cell Migration Assay

The cell migration assay was conducted by using 24-well insert Transwell chambers (Corning Costar, USA). The transfected cells (1 ×10^4^/well) were first suspended in the serum-free medium and then added to the upper chamber. By contrast, the bottom chamber medium contained 10% FBS. After 48 h, the upper chamber was removed, and cells on the filter membrane were wiped off with cotton swabs. Chambers were washed with phosphate-buffered saline (PBS), then fixed in 4% paraformaldehyde for 20 min, washed with PBS, and air-dried. After that, chambers were stained with 0.1% crystal violet solution for 10 min. After washing with distilled water, migrated cells were captured in three random fields under a microscope, and we finally counted the cell number.

### Statistical Analysis

In the present study, all of the data analyses were performed by using SPSS 19.0 software package (SPSS Inc. Chicago, USA). The Student’s *t*-test (two-tailed) and Wilcoxon *t-*test were used to compare the significant differences between the paired and unpaired continuous variables among groups. Pearson χ^2^ or Fisher’s exact test was conducted to analyze the expression or distribution frequency differences of the variables. Logistic regression analysis revealed the association between the two miRNAs expression and the clinical parameters. Kaplan–Meier method and multivariate Cox proportional hazard regression analysis were selected to estimate the potential prognostic value of related indicators. The high-order interactions were assessed between miR-425/576 and clinicopathological variables by using multifactor dimensionality reduction. The data were presented as mean ± standard deviation (SD) or median (quartile). The statistical significance was considered when *p* < 0.05 in all tests.

## Results

### High miR-425 and miR-576 Levels Associated With CRLM

**Figure 1:**
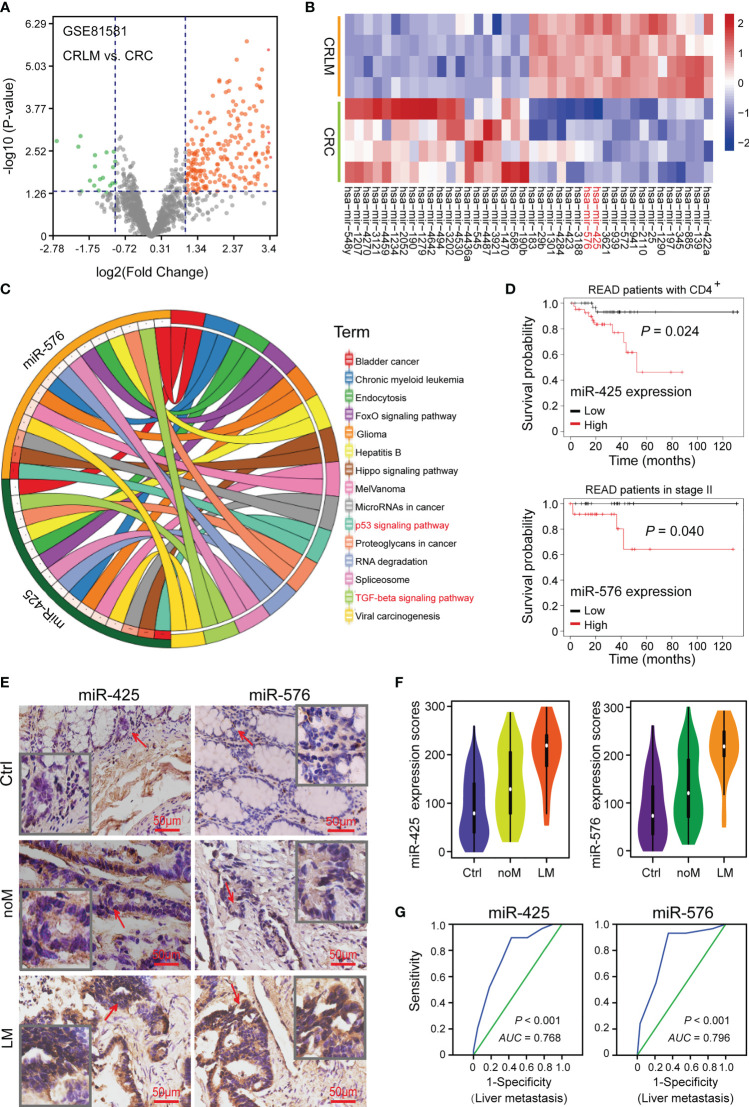
High miR-425 and miR-576 levels associated with CRLM. **(A)** Volcano plot showing the differentially expressed miRNAs between CRLM and primary CRC tissues based on the GSE81581 (*n* = 8) cohort. **(B)** The hierarchical cluster heat map illustrating the TOP20 upregulated and top 20 downregulated miRNAs in CRLM compared with primary CRC (*n* = 4, *p* < 0.05). **(C)** The analysis and visualization of tumor-related pathways in which miR-425 and miR-576 were involved by DIANA TOOL and SangerBox. **(D)** Kaplan–Meier curves showing the association between miR-425 or miR-576 high/low expression and the prognosis of patients with CD4+ or in stage II upon the TCGA database. **(E)** ISH assay determining the cellular localization of miR-425 and miR-576 in CRC tissues with or without liver metastases and the matched adjacent-tumor controls (*n* = 157). Scale bars = 50 μm. **(F)** Violin charts displaying the differences in expression scores of miR-425 and miR-576 in the cohort (*n* = 157). Median (interquartile range) is shown. **(G)** ROC curves determining the liver metastasis-based cutoff scores of miR-425 and miR-576 expression in CRC tissues (*n* = 157).

To explore the vital miRNAs involved in promoting CRLM progression, we analyzed the data from GSE81581 (*n* = 8) cohort to reveal the dysregulated miRNAs between CRLM and primary CRC tissues, and there were 207 upregulated miRNAs and 20 downregulated ones, illustrated in **Figure 1A**. Among them, miR-425 and miR-576 (Log_2_ FC = 3.40 and 3.36, respectively) were further selected for their significant upregulation in CRLM (**Figure 1B**, **Supplementary Figure S1A**, and **Supplementary Table S3**). Furthermore, we investigated the metastasis-related roles that miR-425 and miR-576 collectively played, and P53 and TGF-β signaling pathways were dramatically enriched based on KEGG analysis (**Figure 1C** and **Supplementary Table S4**). Notably, high miR-425 and miR-576 expression shortened the overall survival time of rectum adenocarcinoma (READ) patients with either CD4^+^ or stage II, which both were closely related to CRC metastasis (**Figure 1D**).

In order to verify the abnormal expression of the two miRNAs, we collected tissues from CRC patients (*n* = 157), some of which were with liver metastases (*n* = 29), and matched tumor-adjacent controls. TMA and ISH assays showed that compared with that in controls, the expression of miR-425 and miR-576 was higher in CRC samples, especially in those with liver metastases (**Figures 1E, F** **)**. Thereafter, high area under curve (AUC) landscapes of miR-425 (*AUC* = 0.768, *p* < 0.001) and miR-576 (*AUC* = 0.796, *p* < 0.001) based on liver metastasis and other related risk factors were calculated by drawing ROC curves (**Figure 1G** and **Supplementary Figures S1B, C**), preliminarily determining the convincible diagnostic capacity of them in CRLM. These findings indicate that abnormally high expression of miR-425 and miR-576 plays crucial parts in the CRLM progression.

### Correlation Between miR-425 and miR-576 Expression and CRLM-Related Clinical Variables

**Figure 2:**
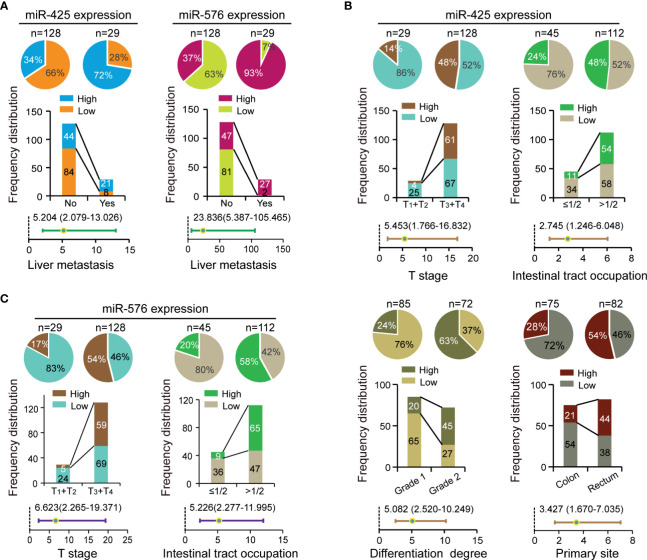
Correlation between miR-425 and miR-576 expression and CRLM-related clinical variables. **(A)** The contribution of miR-425 and miR-576 high expression to liver metastases analyzed by chi-square or Fisher’s exact tests and unconditioned logistic regression. **(B, C)** The correlation of miR-425 **(B)** and miR-576 **(C)** high/low expression with clinicopathological variables analyzed by chi-square or Fisher’s exact tests and unconditioned logistic regression.

To investigate their clinical values in CRLM patients, we analyzed the relationships of miR-425 and miR-576 with environmental factors or pathological variables including age, sex, family history, T stage, N stage, sample size, intestinal tract occupation, differentiation degree, vascular invasion, primary site, and liver metastasis in the selected CRC cohort (*n* = 157). Specifically, the frequency distribution and adjusted regression analyses firstly showed that both miR-425 and miR-576 high expression distributed frequently in the tissues of patients with liver metastases (*p* < 0.05, adjusted OR >1), shown in **Figure 2A** and **Supplementary Table S5**. Furthermore, excessive miR-425 expression was associated with advanced T stage, intestinal tract occupation (>1/2), differentiation degree (grade 2), and primary rectum in CRC (*p* < 0.05, adjusted OR >1), shown in **Figure 2B** and **Supplementary Table S5**. Similarly, increased miR-576 expression was a contributing factor for infiltrating into the serous membrane and extensive intestinal tract occupation (>1/2), shown in **Figure 2C** and **Supplementary Table S5**. The above results suggest that miR-425 and miR-576 promote liver metastasis progression by driving the evolution of the related risk factors in CRC.

To further verify that synergistically overexpressed miR-425 and miR-576 lead to enhancing effects on CRLM, the linear regression analysis first showed a significant positive correlation between them based on our collected 157 samples (*R* = 0.3537, *p* < 0.0001, **Figure S2A**). Thus, after the 157 patients being divided into two subgroups in terms of miR-425 high/low expression, stratification analysis for miR-576 uncovered that there were increases in both the distribution frequency difference and the adjusted OR value of the primary site (**Figure S2B** and **Supplementary Table S6**). Moreover, MDR analysis was performed to reveal that the combination of miR-425 and miR-576 with family history, differentiation degree, and the primary site was the optimal prediction model for its highest cross-validation consistency (CVC) and the least prediction errors in assessing the risk of liver metastasis (**Table 1**). These findings suggested that CRC patients with high miR-425 and miR-576 levels are peculiarly prone to liver metastasis.

**Table 1:** MDR analysis for the prediction of liver metastasis with or without 12 risk factors including high miR-425 and miR-576 expression.

Best interaction models	Liver metastasis		
	Cross-validation consistency	Sign test (*p* ^#^)	Training odds ratio
X2	0.0666	100/100	23.4988 (5.2827–104.5291)
X2,X11	0.0284	97/100	19.1635 (5.4337–67.5858)
X1,X2,X12	0.1184	91/100	28.8576 (6.5056–128.0064)
X1,X2,X10,X12	0.0060	95/100	∞
**X1,X2,X9,X10,X12**	**0.0001**	**98/100**	**∞**
X1,X2,X4,X5,X8,X12	0.4602	79/100	∞
X1,X2,X4,X5,X8,X10,X12	0.0284	96/100	∞
X1,X2,X4,X5,X6,X7,X8,X10	0.3086	92/100	∞
X1,X2,X3,X4,X5,X6,X7,X8,X10	0.8644	94/100	∞
X1,X2,X3,X4,X5,X6,X7,X8,X9,X10	0.9967	96/100	∞
X1,X2,X3,X4,X5,X6,X7,X8,X9,X10,X11	0.9999	95/100	∞
X1,X2,X3,X4,X5,X6,X7,X8,X9,X10,X11,X12	1	100/100	∞

MDR, multifactor dimensionality reduction.

p**^#^** value is for 100-fold permutation test.

The best model with maximum cross-validation consistency and minimum prediction error rate is in bold.

1, miR-425 expression; 2, miR-425 expression; 3, T stage; 4, N stage; 5, sample size (cm); 6, intestinal tract occupation; 7, sex; 8, age (years); 9, family history; 10, differentiation degree; 11, vascular invasion; 12, primary site.

### Impact of miR-425 and miR-576 Expression on Prognosis of CRLM Patients

**Figure 3:**
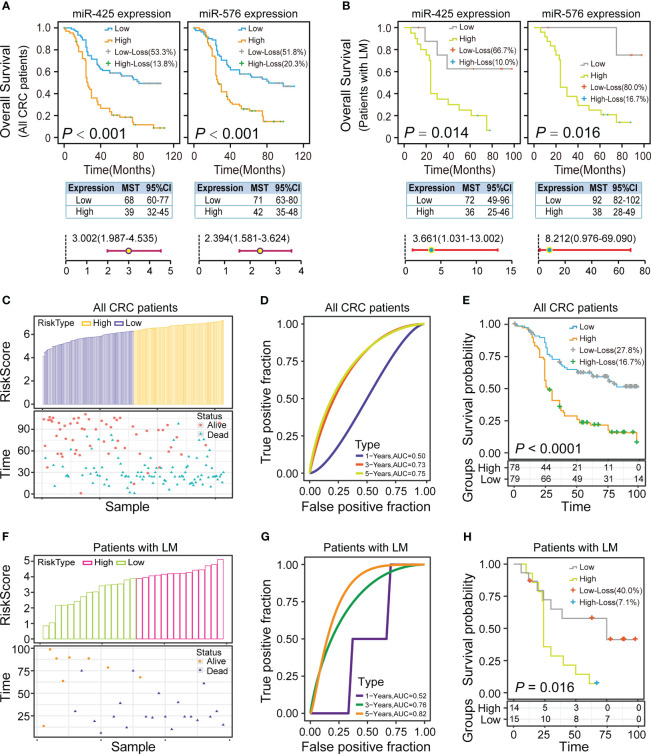
Impact of miR-425 and miR-576 on the prognosis of CRC patients with liver metastases. **(A, B)** Kaplan–Meier curves, log-rank tests, and adjusted Cox regression models showing the association of miR-425 and miR-576 with the OS of the patients with CRC **(A)** and only with CRLM **(B)**. **(C–H)** The risk group, true positive fraction, and proportional hazard of the two-miRNA signature (miR-425 and miR-576) in CRC **(C–E)** especially those in CRLM **(F–H)**, were built and evaluated based on Cox regression and risk score analysis.

To estimate the prognostic application of miR-425 and miR-576 in CRC, we investigated the association of their levels with clinical outcomes through overall and stratification analyses based on these CRC samples (*n* = 157). Totally, the systematic survival analyses revealed that patients with high miR-425 expression had the worse overall survival (OS, *p <* 0.001; high/low expression, MST = 39/68 months, *HR* > 1) and disease-free survival (DFS, *p <* 0.001; high/low expression, MST = 36/67 months, *HR* > 1), and so did those with miR-576 (OS, *P <* 0.001; high/low expression, MST = 42/71 months, HR > 1; DFS, *p <* 0.001, high/low expression, MST = 39/71 months, *HR* > 1), illustrated in **Figure 3A**, **Supplementary Figure S3A**, and **Supplementary Tables S7, S8**.

Then, the stratified survival analysis and MDR analysis were used to explore the synergistic effects of the two miRNAs on CRLM prognosis. Concretely, the results first demonstrated that miR-425 and miR-576 high expression was more significantly related to the OS and DFS of patients with liver metastases (*n* = 29), shown in **Figure 3B**, **Supplementary Figure S3B**, and **Supplementary Tables S7, S8**. Besides, miR-425 low expression effectively alleviated the poor prognosis of patients with low miR-576 expression, and vice versa (**Supplementary Figures S3C, D** and **Supplementary Tables S7, S8**). As expected, the two-factor model consisting of miR-425 and miR-576 could effectively predict the OS states (**Table 2**), while the combination of miR-425 and miR-576 with T stage and differentiation degree was the best model to assess the risk of DFS (**Table 3**). Importantly, we further verified that the assembly of miR-425 and miR-576 was a better prognostic predictor with the high sensitivity in advanced CRC (AUC for 3/5 years = 0.73/0.75) and CRLM (AUC for 3/5 years = 0.76/0.82) by calculating risk scores (**Figures 3C–H** and **Supplementary Figures S3E, F**). These results suggest that the high level of the two-miRNA signature (miR-425 and miR-576) frequently forebodes a poor prognosis in CRC, especially in CRLM.

**Table 2:** MDR analysis for the prediction of OS with or without 13 risk factors including high miR-425 and miR-576 expression.

Best interaction models	OS		
	Cross-validation consistency	Sign test (*p* ^#^)	Training odds ratio
X1	0.3086	100/100	7.1017 (3.1318–16.1040)
**X1,X2**	**0.0284**	**98/100**	**6.7983 (3.2946–14.0279)**
X1,X3,X4	0.9982	69/100	8.4830 (3.9548–18.1959)
X1,X2,X5,X8	0.9940	80/100	12.8208 (5.4133–30.3647)
X1,X2,X5,X8,X10	0.2421	88/100	17.1383 (7.3221–40.1139)
X1,X2,X5,X7,X8,X12	0.9996	68/100	27.1930 (11.0334–67.0200)
X1,X2,X4,X7,X8,X11,X12	0.9940	75/100	55.0337 (20.2416–149.6282)
X1,X2,X3,X5,X7,X7,X8,X12	0.6914	89/100	123.0854 (36.3814–416.4226)
X1,X2,X3,X4,X5,X7,X8,X10,X12	0.9967	97/100	248.9106 (60.5475–1023.2702)
X1,X2,X3,X4,X5,X7,X8,X9,X10,X12	1	84/100	468.4440 (87.2530–2514.9816)
X1,X2,X3,X4,X5,X7,X8,X9,X10,X11,X12	1	86/100	∞
X1,X2,X3,X4,X5,X6,X7,X8,X9,X10,X11,X12	1	100/100	∞
X1,X2,X3,X4,X5,X6,X7,X8,X9,X10,X11,X12,X13	1	100/100	∞

MDR, multifactor dimensionality reduction.

p**^#^** value is for 100-fold permutation test.

The best model with maximum cross-validation consistency and minimum prediction error rate is in bold.

1, miR-425 expression; 2, miR-425 expression; 3, T stage; 4, N stage; 5, sample size (cm); 6, intestinal tract occupation; 7, sex; 8, age (years); 9, family history; 10, differentiation degree; 11, vascular invasion; 12, primary site; 13, liver metastasis.

**Table 3:** MDR analysis for the prediction of DFS with or without 13 risk factors including high miR-425 and miR-576 expression.

Best interaction models	DFS		
	Cross-validation consistency	Sign test (*p* ^#^)	Training odds ratio
X1	0.6914	100/100	8.2789 (3.2341–21.1928)
X1,X2	0.9716	49/100	7.8796 (3.4605–17.9418)
X1,X3,X4	0.0443	99/100	11.9058 (4.8387–29.2949)
**X1,X2,X3,X10**	**0.0018**	**100/100**	**14.5048 (6.0340**–**34.8668)**
X1,X2,X3,X4,X10	0.9334	73/100	20.0722 (8.2334–48.9336)
X1,X2,X3,X4,X10,X12	0.9334	75/100	48.6573 (14.4132–164.2616)
X1,X2,X4,X5,X6,X8,X12	0.8644	86/100	∞
X1,X2,X4,X5,X6,X8,X10,X12	0.7579	97/100	∞
X1,X2,X3,X4,X5,X6,X8,X10,X12	0.9892	84/100	∞
X1,X2,X3,X4,X5,X7,X8,X9,X10,X12	0.9895	97/100	∞
X1,X2,X3,X4,X5,X6,X7,X8,X9,X10,X12	1	100/100	∞
X1,X2,X3,X4,X5,X6,X7,X8,X9,X10,X12,X13	1	98/100	∞
X1,X2,X3,X4,X5,X6,X7,X8,X9,X10,X11,X12,X13	1	100/100	∞

MDR, multifactor dimensionality reduction.

p**^#^** value is for 100-fold permutation test.

The best model with maximum cross-validation consistency and minimum prediction error rate is in bold.

1, miR-425 expression; 2, miR-425 expression; 3, T stage; 4, N stage; 5, sample size (cm); 6, intestinal tract occupation; 7, sex; 8, age (years); 9, family history; 10, differentiation degree; 11, vascular invasion; 12, primary site; 13, liver metastasis.

### PTEN Identified as the Target of miR-425 and miR-576 Facilitating CRLM

**Figure 4:**
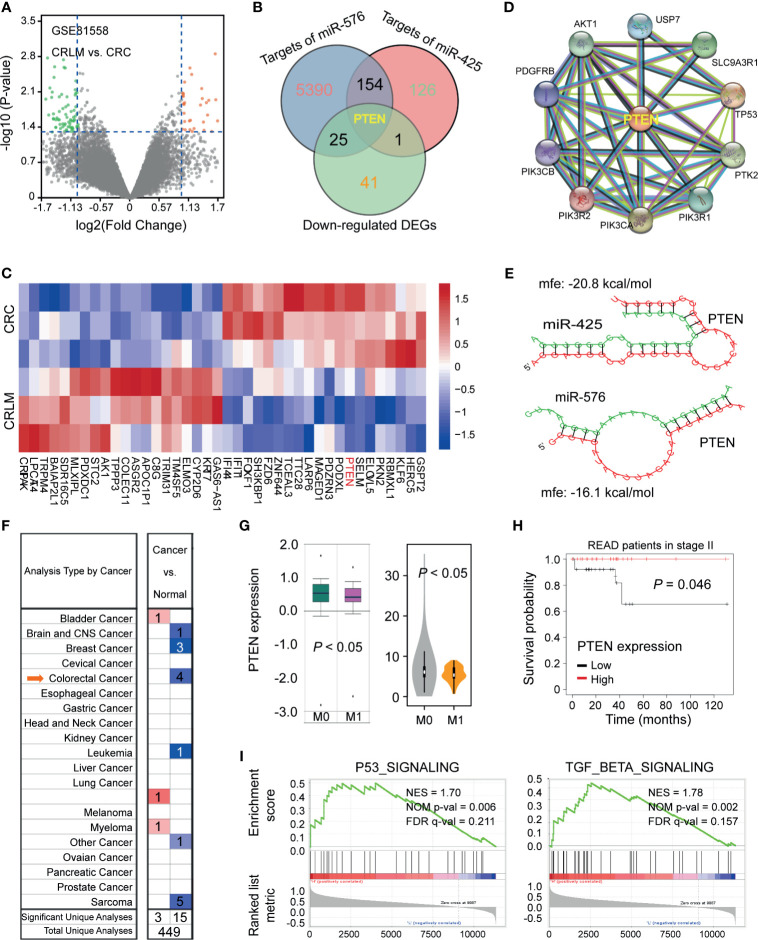
PTEN identified as the target of miR-425 and miR-576 facilitating CRLM. **(A)** Volcano plot showing the differentially expressed mRNAs between CRLM and primary CRC tissues based on the GSE81558 (*n* = 6) cohort. **(B)** Taking an intersection of mRNAs among those down-regulated in GSE81558 (*n* = 6) and targeted by miR-425 and miR-576 *via* Venn diagram. **(C)** The hierarchical cluster heat map illustrating the top 20 upregulated and top 20 downregulated mRNAs in CRLM compared with primary CRC (*n* = 4, *p* < 0.05). Red in heat map denotes upregulation. Blue denotes downregulation. **(D)** STRING sever predicting the protein–protein interaction networks related to PTEN. **(E)** Evaluating the sites and MFEs of miR-425/miR576 binding to *PTEN* 3′UTR through *RNAhybrid.* **(F)** The distribution of *PTEN* levels in various tumors, including CRC, compared with the normal by searching Oncomine (https://www.oncomine.org/). **(G)** The expression differences of *PTEN* between the metastasis and the blank groups in CRC samples based on the TCGA database. **(H)** Kaplan–Meier curves showing the association between *PTEN* high/low expression and the prognosis of patients in stage II upon the TCGA database. **(I)** GSEA analysis enriching the signaling pathways in which high *PTEN* expression participated.

To clarify the meaningful downstream target of miR-425 and miR-576 mediating liver metastasis and poor prognosis, we analyzed the DEGs between CRC tissues and matched tumor-adjacent controls upon GSE81558 (*n* = 6) dataset by drawing a volcano plot (**Figure 4A**). Thereafter, *PTEN* was enriched for its dramatic downregulation in CRLM and potential to be bound by miR-425 and miR-576 through Venn diagram (**Figure 4B**). Coincidentally, the heat map cluster showed that *PTEN* was most significantly decreased in CRLM (Log_2_ FC = 1.63), illustrated in **Figure 4C** and **Supplementary Table S9**. Furthermore, the key proteins interacting with PTEN in cancer were present by further searching STRING (**Figure 4D**). Of note, we explored the complimentary domain and binding sites between *PTEN* 3′UTR and miR-425 (+2928 to +2934) or miR-576 (+4371 to +4378). Meanwhile, the cogent MFEs between the pairing sequences were calculated as −20.8 and −16.1 kcal/mol, respectively (**Figure 4E**). Further analysis revealed that *PTEN* expression was significantly downregulated in several types of cancer based on TCGA database (**Figure 4F**), and the lower levels of PTEN expression were prone to tumor metastasis and poor prognosis in CRC (**Figures 4G, H** **)**. Moreover, consistent with miR-425 and miR-576, PTEN was confirmed to positively participate in P53 and TGF-β signaling pathways, which played meaningful parts in tumor metastasis based on GSE62321 (*n* = 26) dataset (**Figure 4I**). These findings indicate that downregulated PTEN acts as the shared target of miR-425 and miR-576 in CRLM, thereby leading to unfavorable clinical outcomes.

### Effect of miR-425 and miR-576 on Tumor Metastasis by Inhibiting PTEN-P53/TGF-β Axis in CRC Cells

**Figure 5:**
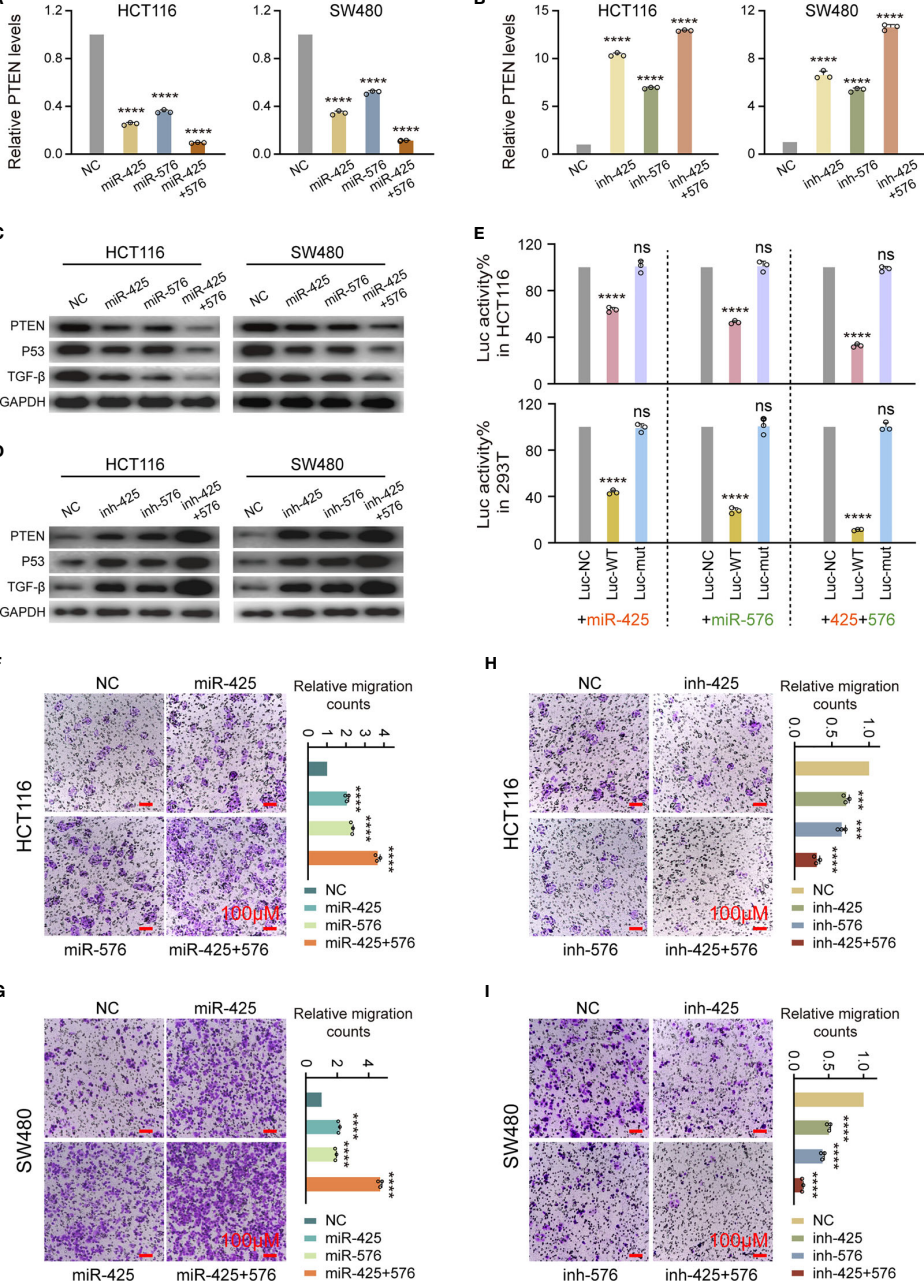
Effect of miR-425 and miR-576 on tumor metastasis by inhibiting PTEN-P53/TGF-β axis in CRC Cells. **(A, B)** The levels of *PTEN* mRNA after miR-425/miR-576 overexpression **(A)** or knockdown **(B)** in HCT116 and SW480 cells detected by qPCR assay. **(C, D)** The changes in PTEN protein and P53 and TGF-β upon interfering miR-425 and/or miR-576 expression in HCT116 and SW480 cells detected by Western blot assay. **(E)** Dual-Luciferase reporter assay identifying the specific binding activities of *PTEN* 3′UTR with miR-425/miR-576 in HCT116 and HEK293T cells. **(F–I)** The cell migration abilities in CRC cells treated with mimics and inhibitors of miR-425 and/or miR-576 detected by cell migration assay. All tests were performed at least three times. Data were expressed as mean ± SD (ns, *p* > 0.05; ***p < 0.001; ****p < 0.0001).

Next, we demonstrated that miR-425 and miR-576 induced the occurrence of metastasis from CRC by negatively regulating PTEN-P53/TGF-β axis identified *via* loss- and gain-function experiments at the cytological level. Concretely, qPCR assay identified that the overexpression of the two miRNAs caused substantial inhibition of *PTEN* mRNA expression in both HCT116 and SW480 cells (**Figure 5A**). Conversely, miR-425 and miR-576 depletion significantly enhanced the expression of *PTEN* mRNA in such two types of CRC cells (**Figure 5B**). Importantly, the changes in the PTEN protein were proved consistent by using Western blotting, so were in the P53 and TGF-β (**Figures 5C, D** and **Supplementary Figures S4A, B**). Since a relatively higher affinity was predicted among these indicators, we subsequently performed the luciferase intensity detection after cotransfected with PTEN 3′UTR-WT/Mut luciferase reporter and miR-425 and/or miR-576 mimics in HCT116 and HEK293T cells. As expected, the results showed that miR-425 and/or miR-576 dramatically suppressed the expression intensity of WT luciferase reporters (**Figure 5E**); however, no significant alteration was observed on the Mut ones, suggesting a direct bonding pattern between *PTEN* 3′UTR and miR-425 and/or miR-576 (**Figure 5E**). Furthermore, ectopic miR-425 and miR-576 expression observably facilitated the migration abilities of HCT116 and SW480 cells (**Figures 5F, G** **)** and vice versa (**Figures 5H, I** **)**. Collectively, these results demonstrated that miR-425 and miR-576, directly targeting PTEN-mediated P53/TGF-β signaling pathways, are involved in the promotion of cell migration in the course of CRLM.

### Immune Analysis of miR-425/miR-576-PTEN Axis for CRLM

**Figure 6:**
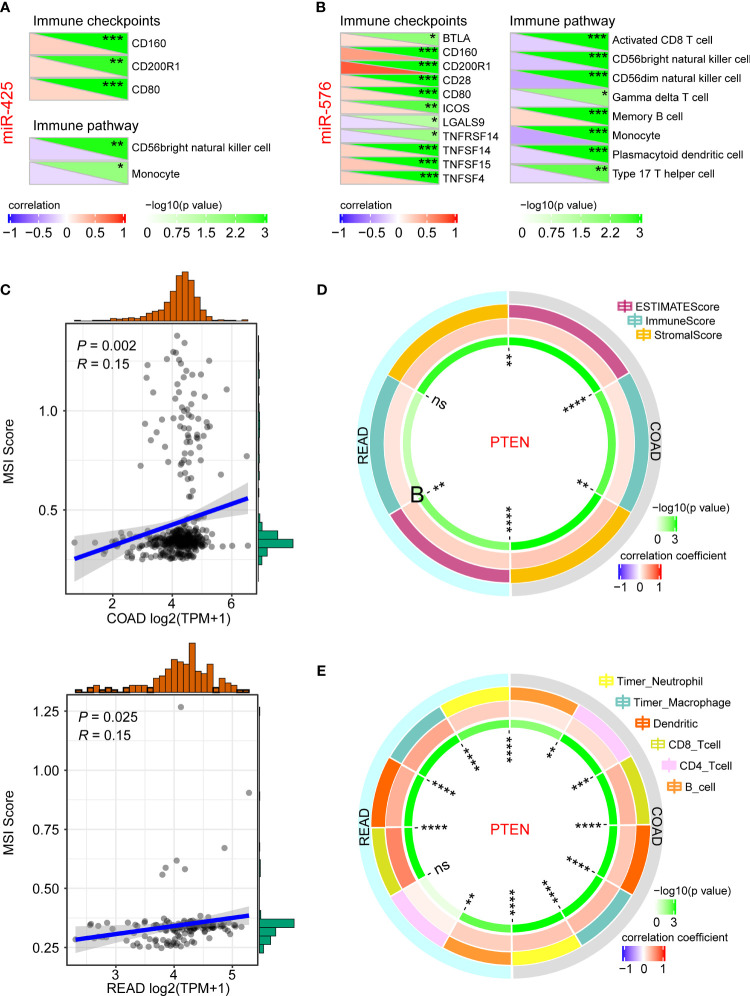
Immune analysis of miR-425/miR-576 and PTEN for CRLM. **(A, B)** The association of miR-425 **(A)** and miR-576 **(B)** with immune checkpoints and immune pathways in CRC progression. **(C–E)** The correlation of *PTEN* with MSI score **(C)**, TME **(D)**, and immune cell infiltration **(E)** in CRC progression (*p < 0.05; **p < 0.01; **p < 0.001; ****p < 0.001).

To further investigate their crucial roles in immune regulation of CRLM initiation and development, we first analyzed the association of miR-425 and miR-576 with the related indicators. As a result, miR-425 was closely related to immune checkpoints such as CD160, CD200R1, and CD80, and immune pathways including CD56bright natural killer cell and monocyte in CRC progression (**Figure 6A**). Similarly, there were significant relationships of miR-425 with the checkpoints (BTLA, CD160/200R1/28/80, COS, LGALS9, TNFRSF14, and TNFSF14/15/4) and pathways (activated CD8 T cell, CD56bright/CD56dim natural killer cell, gamma delta T cell, memory B cell, monocyte, plasmacytoid dendritic cell, and type 17 T helper cell), illustrated in **Figure 6B**. Moreover, we further discovered that tumor suppressor PTEN was positively correlated with MSI (**Figure 6C**) and TME (ESTIMATE Score, Immune Score, and Stromal Score) and immune cell infiltration (neutrophil, macrophage, dendritic, CD8_Tcell, and B_cell), which clarified the effects of PTEN on regulating CRLM based on the perspective of immune (**Figures 6D, E** **)**. The results above indicate that miR-425/miR-576-mediated PTEN inhibition promotes the CRLM process by triggering immune escape.

## Discussion

At present, the majority of patients with CRC blindly reach a severe advanced pathological stage with liver metastases due to a lack of evaluative biomarkers (39). Furthermore, although the first-line surgical and chemotherapy regimens are generally accepted, emerging immunotherapies have shown significant improvement in patient survival, suggesting the urgency to explore immune-based targets for CRLM treatment (40). In our study, the significantly upregulated miR-425 and miR-576, targeting PTEN, were identified as key factors in CRLM.

It has been reported that miRNAs were proposed as biomarkers to assess tumorigenesis and survival in CRLM, on account of not only their expression differences in tumors but also their abilities to mediate the rapid progression and adverse outcomes (41–43). It is noteworthy that miR-425 was identified as a biomarker in a wide variety of tumors such as gastric cancer, prostate cancer, and liver cancer (44–46), while miR-576 played predictive roles in bladder cancer, breast cancer, and nonmelanoma skin cancer (14, 47, 48). However, the potential of miR-425 and miR-576 in CRLM might not have been considered. In our study, we first noticed miR-425 and miR-576 for their abnormally high expression in liver metastases based on the GSE81581 (*n* = 8) dataset containing sample information on primary CRC and CRLM. Besides, the two miRNAs were proven to shorten the survival of patients with either CD4^+^ or stage II in CRC, which both were closely related to tumor metastasis (49, 50). These findings preliminarily demonstrate the ability of miR-425 and miR-576 to predict and evaluate liver metastasis. To provide more evidence that miR-425 and miR-576 acted as potential biomarkers, we further performed ISH assay on our included CRC samples (n = 157). Statistical analysis results showed that upregulated miR-425 and miR-576 levels were easier to appear in samples with liver metastases, which showed high predictive sensitivities by drawing ROC curves. In addition, miR-425 high expression was associated with advanced T stage, intestinal tract occupation (>1/2), low differentiation, and primary rectum. Analogously, miR-576 high expression was a contributing factor for the infiltration into the serous membrane and extensive intestinal tract occupation (>1/2). What is more, high miR-425 or miR-576 expression was associated with a poor prognosis of CRLM patients. As we have seen, all of the above based on sufficient samples clearly verified the existed clinical relevance of miR-425 and miR-576.

More interestingly, a multitude of studies attested that muti-miRNA signature tended to show more convincing forecasting ability for tumor occurrence and progression (51–53). For instance, the two-factor model including miR-33a-5p and miR-128-3p in whole blood acted as a potential biomarker for early diagnosis in lung cancer (51); the combination of three miRNAs (miR-141, miR-21, and miR-375) played roles in recognizing prostate cancer (52), and the 6-miRNA signature (miR-15b-5p, miR-18a-5p, miR-29a-3p, miR-335-5p, miR-19a-3p, and miR-19b-3p) in serum from CRC screening participants was identified as noninvasive biomarker for advanced adenoma and CRC detection (53). Thus, on the premise of the certified positive correlation of expression between miR-576 and miR-425, we further spied that the combination of miR-425 and miR-576 with family history, differentiation, and the primary site was the optimal signature to assess the risk of liver metastasis. Besides, systematic survival analyses revealed that the prognostic model based on miR-425 and miR-576, with high sensitivities, effectively evaluated clinical outcomes in CRC, especially in CRLM. In brief, we constructed a novel two-miRNA panel to predict the liver metastases and survival states of patients with CRC.

As we all know, it is usual that most miRNAs play the regulatory roles indirectly by binding to and degrading the target mRNAs. Given this molecular mechanism, we further confirmed that PTEN, significantly downregulated in CRLM, was the cosuppression target of miR-425 and miR-576 collaboratively acting as the tumor promoters. In addition to the negative regulatory patterns in CRC cells, we identified the complimentary domain between *PTEN* 3′UTR and miR-425 (+2928 to +2934) or miR-576 (+4371 to +4378) and calculated the cogent MFEs between them (−20.8 and −16.1 kcal/mol), in some detail. To further clarify that, we verified their direct binding interaction in HCT116 and HEK293T cells by Dual-Luciferase reporter assay. Besides, it is remarkable that miR-425 and miR-576 overexpression has been shown to promote the migration ability of HCT116 and SW480 cells by Transwell migration assay and vice versa. Similarly, miR-425 was discovered to play positive roles in not only promoting tumorigenesis and metastasis through activating CTNND1-mediated β-catenin pathway in CRC but also facilitating CRC chemoresistance by targeting PDCD10 (15, 54). Of note, it has been reported that exosomal miR-425 induced liver metastasis by regulating PTEN through activation of the PI3K/Akt signaling pathway (55). Herein, however, P53 and TGF-β signaling pathways were the indispensable links that miR-425/PTEN axis regulated CRLM, which was actually validated in CRC cells. As for miR-576, our study was the first to reveal its potential as a biomarker for liver metastasis and the regulation mechanism involving downstream PTEN-P53/TGF-β signaling axis in the process of CRC cells undergoing liver metastasis.

Recently, with the rise in the anti-miRNAs strategy, to explore more miRNAs involved in crucial tumor-related pathways as prospective targets has become a shared purpose. Although the critical roles of miRNAs have always been manifested for CRC (56), even CRLM (57), no specific miRNA-based strategy has been reported yet, which indicated that the mining miRNAs with potential for approval was an urgent mission. Besides, increasing evidence indicated that immunotherapy has become a powerful clinical strategy for treating cancer (58). However, the key challenges in the broad implementation of such therapy for cancer remains to be the serious adverse effects and poor treatment response due to the complex modulation and components of the immune system, which suggests that it is particularly important to clarify the molecular mechanism of immunotherapeutic targets (59). Our results demonstrated that upregulation of miR-425 and miR-576 was closely related to disease progression and shortened survival in CRLM, and their shared target PTEN not only suppressed P53/TGF-β signaling pathways but also was positively related to MSI, TME, and immune cell infiltration. At present, PTEN has been confirmed to function at the interface between cancer and tumor immune microenvironment (TIME) (31), which plays crucial roles in immune biological processes and the response of cancer to immunotherapy (60); however, its interaction network remains to be elucidated. Thus, on the basis of illustrating the signaling axis, we first proposed that miR-425 and miR-576 were promising potential immunotherapeutic targets for the individualized anti-miRNAs treatment of CRLM patients.

In conclusion, elevated miR-425 and miR-576 were associated with tumorigenesis and worse outcomes and negatively involved in PTEN-P53/TGF-β-immune function axis in CRLM. Therefore, our work is beneficial to better understanding the potential applications of miRNA–mRNA to provide an immediate cue for potential biomarkers and targets in CRLM prevention and immunotherapy.

## Data Availability Statement

The datasets presented in this study can be found in online repositories. The names of the repository/repositories and accession number(s) can be found in the article/**Supplementary Material**.

## Ethics Statement

The studies involving human participants were reviewed and approved by The Medical Ethics Committee of China Medical University. Written informed consent to participate in this study was provided by the participants’ legal guardian/next of kin.

## Author Contributions

HW, YL and MW conceived and designed the project. XH, QC, KL and BF designed and supervised experiments conducted in the laboratories. XH, QC, HG and YC performed experiments and/or data analyses. HW, MW, YL, and HZ contributed reagents/analytic tools and/or grant support. HW, YL, MW, and XH wrote the paper. All authors contributed to the article and approved the submitted version.

## Funding

This work was supported by grants from the National Natural Science Foundation of China (81872905, 31828005, and 81673475), National Natural Science Foundation of China and Liaoning joint fund key program (U1608281), Liaoning Revitalization Talents Program (XLYC1807155), Shenyang S&T Projects (20-204-4-22), the Science and Technology Innovative Foundation for Young and Middle-aged Scientists of Shenyang City (RC200382), Shenyang High Level Talent Innovation and Entrepreneurship Team (2019-SYRCCY-B-01), Key R&D Guidance Plan Projects in Liaoning Province (2019JH8/10300011), and Major Special S&T Projects in Liaoning Province (2019JH1/10300005).

## Conflict of Interest

The authors declare that the research was conducted in the absence of any commercial or financial relationships that could be construed as a potential conflict of interest.

## Publisher’s Note

All claims expressed in this article are solely those of the authors and do not necessarily represent those of their affiliated organizations, or those of the publisher, the editors and the reviewers. Any product that may be evaluated in this article, or claim that may be made by its manufacturer, is not guaranteed or endorsed by the publisher.

## Abbreviations

CRC, colorectal cancer; READ, rectum adenocarcinoma; CRLM, colorectal liver metastasis; miRNA, microRNA; miR-425, hsa-miR-425-5p; miR-576, hsa-miR-576-3p; TNBC, triple-negative breast cancer; CCA, cholangiocarcinoma; 3′UTR, 3′-untranslated region; PTEN, phosphatase and tensin homologue deleted on chromosome 10; DEG, differentially expressed gene; TMA, tissue microarray; ISH, *in situ* hybridization; DFS, disease-free survival; OS, overall survival; MSI, microsatellite instability; TME, tumor microenvironment.
