# Supplementary material for: Identification of Target PTEN-Based miR-425 and miR-576 as Potential Diagnostic and Immunotherapeutic Biomarkers of Colorectal Cancer With Liver Metastasis
Source: Front Oncol. 2021 Aug 19;11:657984. doi: 10.3389/fonc.2021.657984 (PMC8418231; doi:10.3389/fonc.2021.657984)
Supplement: Supplementary file 1 [file DataSheet_1.doc]

**Identification of target PTEN-based miR-425 and miR-576 as potential diagnostic and immunotherapeutic biomarkers of colorectal cancer with liver metastasis**

**Supplemental Information Outline**

- **Supplemental data**
- **Figure S1. High miR-425 and miR-576 levels associated with CRLM**
- **Figure S2. Correlation of miR-425 and miR-576 expression with CRLM-related clinical variables**
- **Figure S3. Correlation of miR-425 and miR-576 expression with the prognosis of patients with CRLM**
- **Figure S4. Effect of miR-425 and miR-576 on tumor metastasis by inhibiting PTEN-P53/ TGF-β axis in CRC Cells**
- **Table S1. Clinicopathological variables in CRC patients (n=157)**
- **Table S2. Probes used in situ hybridization (ISH) assay**
- **Table S3. Data of miRNAs cluster in Heatmap analysis**
- **Table S4. The pathways in which miR-425 and miR-576 were involved**
- **Table S5. Correlation analysis between miR-425/miR-576 and clinical variables in CRC patients (n=157)**
- **Table S6. [Stratified](../../../../F:/Program%20Files/Youdao/Dict/8.9.4.0/resultui/html/index.html#/javascript:;) correlation analysis between miR-425/miR-576 and clinical variables in CRC patients (n=157)**
- **Table S7. The prognostic information of included CRC patients (n=157)**
- **Table S8. Multivariate Cox regression analysis of the [correlation](../../../mac/AppData/Local/youdao/dict/Application/8.9.3.0/resultui/html/index.html#/javascript:;) between *SENCR* and survival (DFS and OS) in CRC patients (n=157)**
- **Table S9. Data of mRNAs cluster in Heatmap analysis**

**
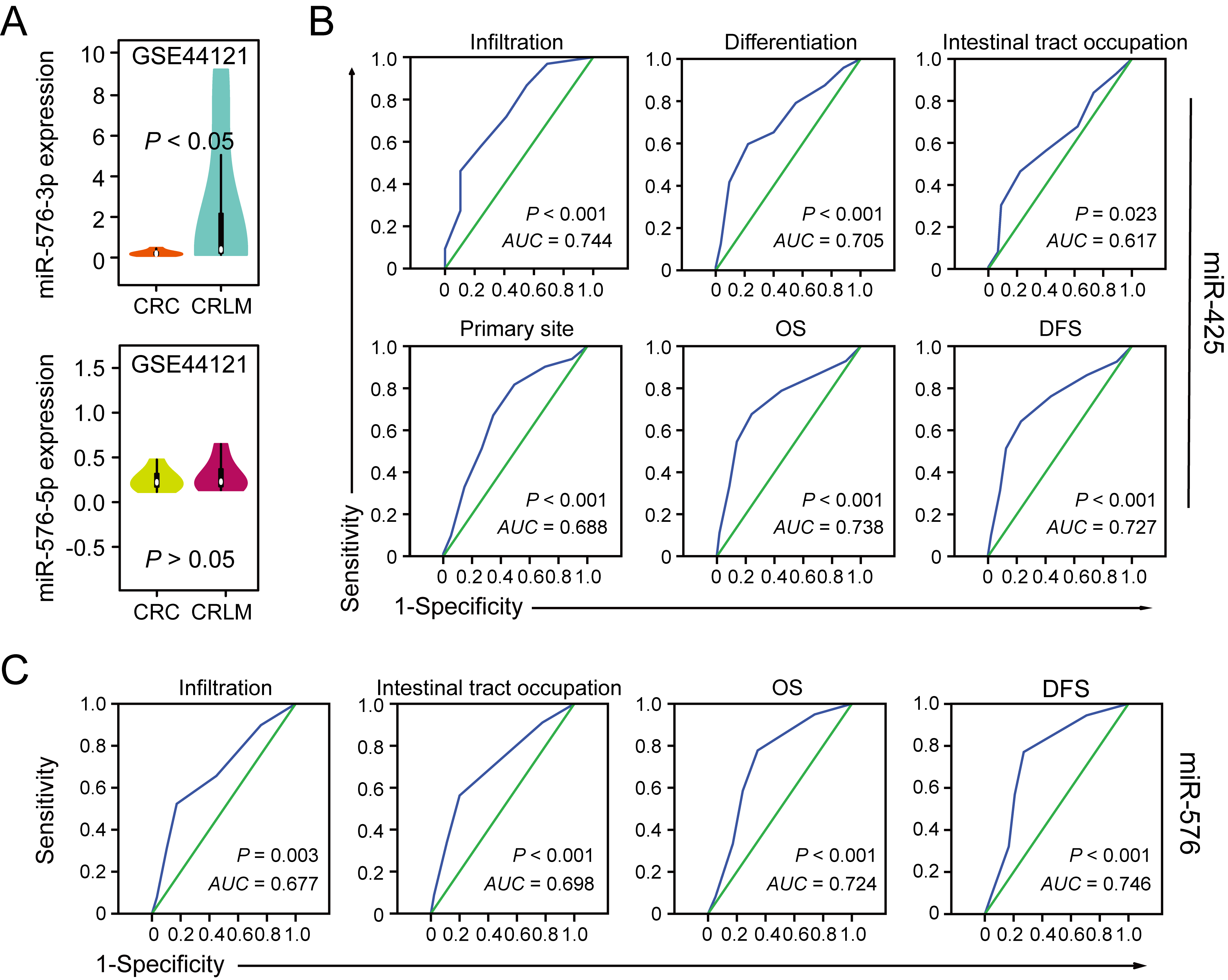
**

**Figure S1. High miR-425 and miR-576 levels associated with CRLM**

**(A)** The expression levels of miR-576-3p and miR-576-5p in CRLM compared with primary CRC tissues. **(B and C)** ROC curves of miR-425 (B) and miR-576 (C) expression based on included [variable](../../../../D:/%25E8%25BD%25AF%25E4%25BB%25B6/youdao/Dict/8.9.5.0/resultui/html/index.html#/javascript:;)s in CRC tissues (n=157).

**
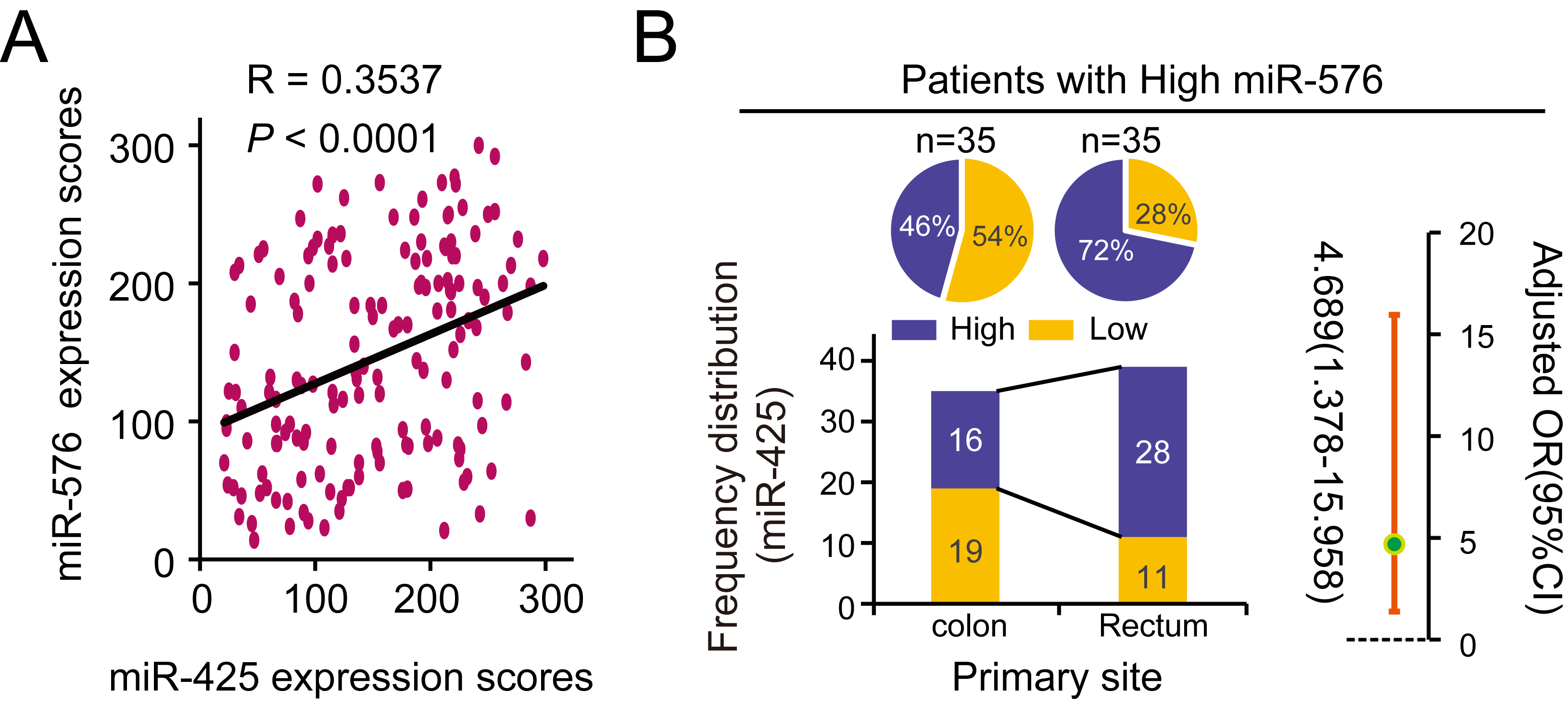
**

**Figure S2. Correlation between miR-425 and miR-576 expression and CRLM-related clinical variables**

**(A)** Linear regression analysis of expression between miR-425 and miR-576 upon our included CRC samples cohort (n = 157). **(B)** The [stratified](../../../../F:/Program%20Files/Youdao/Dict/8.9.4.0/resultui/html/index.html#/javascript:;) correlation of miR-425 high/low expression with the primary site analyzed by Chi-square or [Fisher's exact test](http://www.so.com/link?m=amOc/yl3UD4OLSGP/mEzNdsu919KXT0cXLoQVmqOsZ0c2fwFL4xK49fVzSZ8WTWlYESvf5I/BzqgO6Qp/YY86CknwXGqGtKs2OPsBMx4D/xmOBeYBflciMXbJHZUpeY7Rmrj9QerMpzsfXsLX4z0yzn+CN08GXDyoTggyPnRNLWtkVGgAgWFUPzThLgnYoBCr/AbfBZ/yFFM=)s and [unconditioned](#/javascript:;) logistic regression.

**
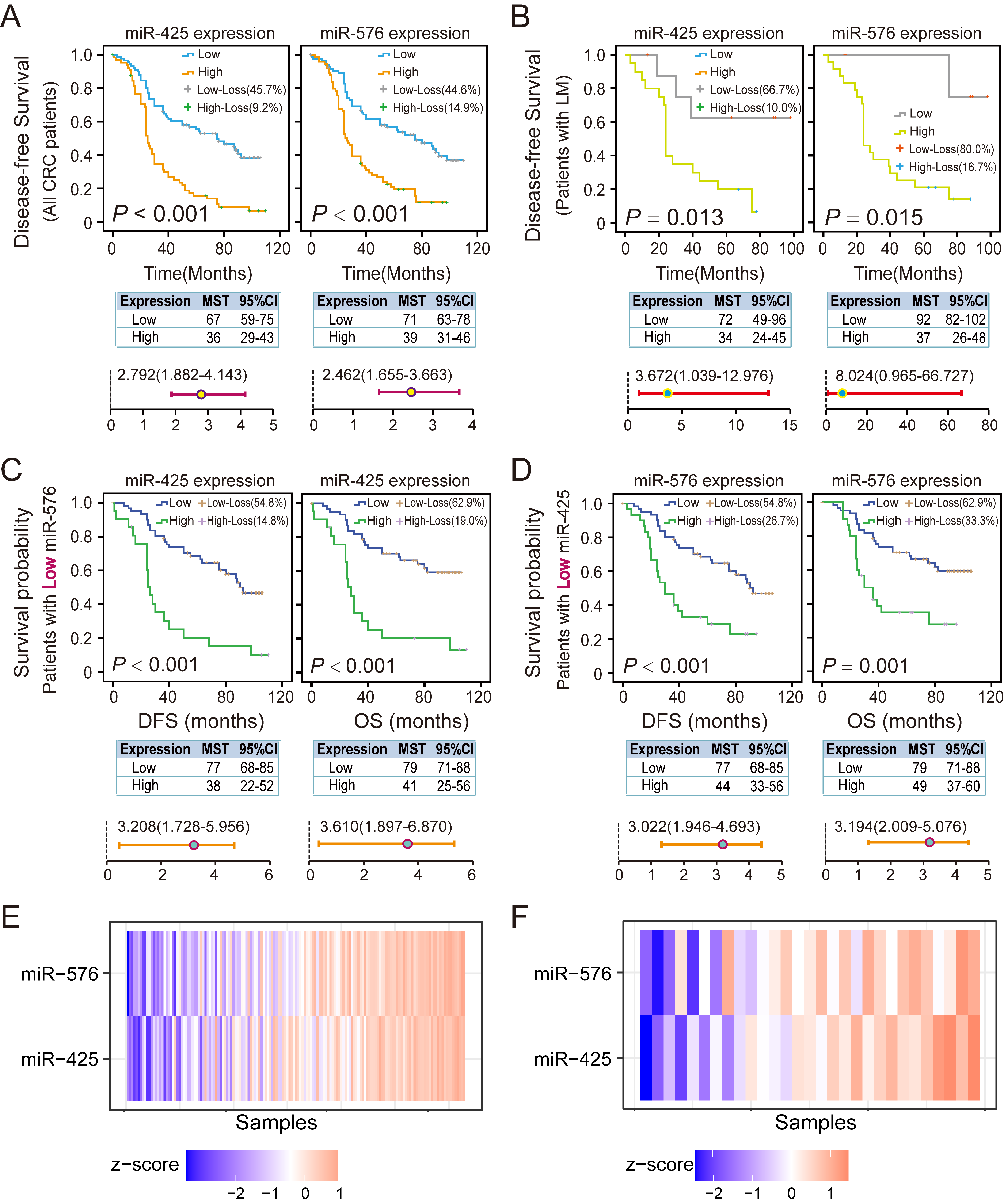
**

**Figure S3. Correlation of miR-425 and miR-576 expression with the prognosis of patients with CRLM**

**(A and B)** Kaplan-Meier curves, Log-rank tests, and adjusted COX [regression](#/javascript:;) models showing the association of miR-425 and miR-576 with the DFS of the patients with CRC (A) and only with CRLM (B). **(C and D)** The miR-425/miR-576-based [stratification](#/javascript:;) of Kaplan-Meier curves, Log-rank tests, and COX [regression](#/javascript:;) models showing their association with the DFS and OS of CRC patients. **(E and F)** Z-scores of the two-miRNA [signature](../../../../D:/software/youdao/8.9.6.0/resultui/html/index.html#/javascript:;) (miR-425 and miR-576) in CRC (E) especially those in CRLM (F) built and evaluated by COX [regression](#/javascript:;) and risk score analyses.


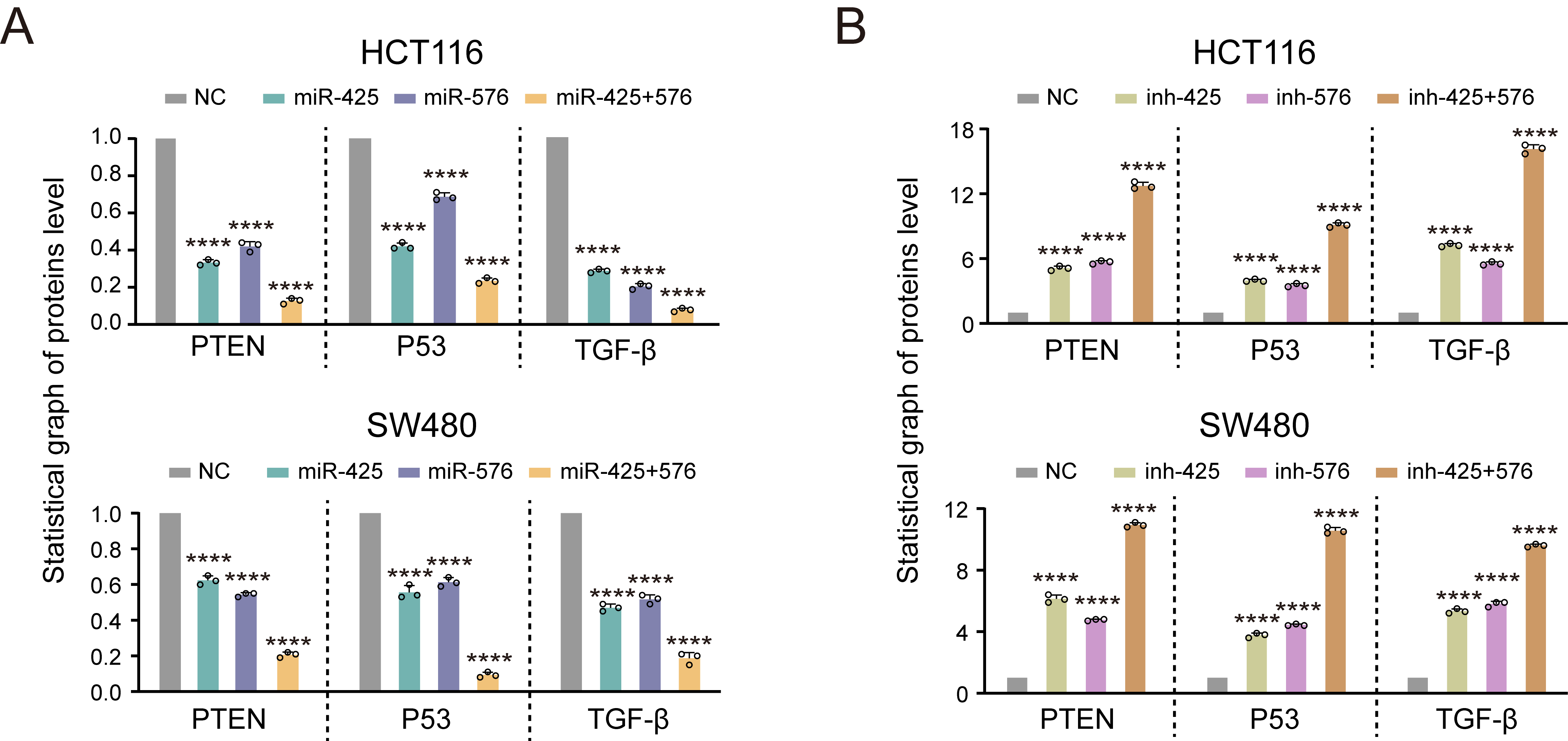


**Figure S4. Effect of miR-425 and miR-576 on tumor metastasis by inhibiting PTEN-P53/ TGF-β axis in CRC Cells**

**(A and B)** The [statistical](../../../../D:/software/youdao/8.9.6.0/resultui/html/index.html#/javascript:;) [diagram](../../../../D:/software/youdao/8.9.6.0/resultui/html/index.html#/javascript:;)s of the changes in PTEN protein, as well as P53 and TGF-β upon interfering miR-425 and/or miR-576 expression in HCT116 and SW480 cells based on Western blot assay.

**Table S1. Clinicopathological variables in CRC patients (n=157)**

|  | **No. of Cases** | **%** |
| --- | --- | --- |
| **Total no.** | 157 | 100 |
| **Average Age [range](years)** | 64[17-88] |  |
| **Age (years)** |  |  |
| ≤64 | 77 | 49.0 |
| >64 | 80 | 51.0 |
| **Sex** |  |  |
| Male | 87 | 55.4 |
| Female | 70 | 44.6 |
| **Family History** |  |  |
| No | 143 | 91.1 |
| Yes | 14 | 8.9 |
| **T stage** |  |  |
| T_1_+T_2_ | 29 | 18.5 |
| T_3_+T_4_ | 128 | 81.5 |
| **N stage** |  |  |
| No | 69 | 43.9 |
| Yes | 88 | 56.1 |
| **Sample size (cm)** |  |  |
| ≤6 | 87 | 55.4 |
| >6 | 70 | 44.6 |
| **Intestinal tract occupation** |  |  |
| ≤1/2 | 45 | 28.7 |
| >1/2 | 112 | 71.3 |
| **Differentiation degree** |  |  |
| Grade 1 | 85 | 54.1 |
| Grade 2 | 72 | 45.9 |
| **Vascular invasion** |  |  |
| No | 141 | 89.8 |
| Yes | 16 | 10.2 |
| **Primary site** |  |  |
| Colon | 75 | 47.8 |
| Rectum | 82 | 52.2 |
| **Liver metastasis** |  |  |
| No | 128 | 81.5 |
| Yes | 29 | 18.5 |
| **DFS** |  |  |
| Negative | 49 | 31.2 |
| Positive | 108 | 68.8 |
| **OS** |  |  |
| Survival | 59 | 37.6 |
| Death | 98 | 62.4 |
| **Follow-up** |  |  |
| DFS (Months) | 41.97[1-110] |  |
| OS (Months) | 53.75[6-110] |  |

*Abbreviations：****DFS****, Disease-free survival;* ***OS****, Overall survival*

**Table S2. Probes used in situ hybridization (ISH) assay**

| **Digoxin-labeled probe** | **Sequence (5'-3')** |
| --- | --- |
| Has-miR-425-5p | TCAAC GGGAG TGATC GTGTC ATT |
| Has-miR-576-3p | GATTC CAATT TTTCC ACATC TT |

**Table S3. Data of miRNAs cluster in Heatmap analysis**

| **UP-regulated miRNA** | **Fold Change** | **Down-regulated miRNA** | **Fold Change** |
| --- | --- | --- | --- |
| **hsa-mir-425** | 3.40 | hsa-mir-1234 | 2.78 |
| **hsa-mir-576** | 3.36 | hsa-mir-494 | 2.71 |
| hsa-mir-941 | 3.35 | hsa-mir-4270 | 2.08 |
| hsa-mir-3188 | 3.33 | hsa-mir-548y | 2.02 |
| hsa-mir-572 | 3.33 | hsa-mir-4459 | 2.02 |
| hsa-mir-29b | 3.31 | hsa-mir-1279 | 1.75 |
| hsa-mir-423 | 3.31 | hsa-mir-4530 | 1.63 |
| hsa-mir-1290 | 3.30 | hsa-mir-2052 | 1.62 |
| hsa-mir-345 | 3.30 | hsa-mir-190b | 1.61 |
| hsa-mir-139 | 3.28 | hsa-mir-4642 | 1.51 |
| hsa-mir-422a | 3.25 | hsa-mir-4487 | 1.46 |
| hsa-mir-4284 | 3.24 | hsa-mir-1470 | 1.39 |
| hsa-mir-183 | 3.17 | hsa-mir-3121 | 1.28 |
| hsa-mir-25 | 3.17 | hsa-mir-4436a | 1.28 |
| hsa-mir-1301 | 3.15 | hsa-mir-3202 | 1.19 |
| hsa-mir-885 | 3.15 | hsa-mir-3921 | 1.12 |
| hsa-mir-197 | 3.15 | hsa-mir-586 | 1.09 |
| hsa-mir-2110 | 3.14 | hsa-mir-545 | 1.08 |
| hsa-mir-939 | 3.11 | hsa-mir-1207 | 1.05 |
| hsa-mir-3621 | 3.08 | hsa-mir-190 | 1.03 |

**Table S4. The pathways in which miR-425 and miR-576 were involved**

| **KEGG pathway** | **p-value** | **#genes** | **#miRNAs** |
| --- | --- | --- | --- |
| p53 signaling pathway | 2.17E-11 | 24 | miR-425, miR-576 |
| Hippo signaling pathway | 7.03E-05 | 22 | miR-425, miR-576 |
| Protein processing in endoplasmic reticulum | 0.00080091 | 26 | miR-425, miR-576 |
| MicroRNAs in cancer | 0.000892963 | 30 | miR-425, miR-576 |
| Viral carcinogenesis | 0.010949391 | 21 | miR-425, miR-576 |
| Melanoma | 0.011674541 | 10 | miR-425, miR-576 |
| Glioma | 0.016695112 | 10 | miR-425, miR-576 |
| Chronic myeloid leukemia | 0.016695112 | 12 | miR-425, miR-576 |
| RNA degradation | 0.025165734 | 12 | miR-425, miR-576 |
| Spliceosome | 0.026104406 | 15 | miR-425, miR-576 |
| FoxO signaling pathway | 0.028873667 | 19 | miR-425, miR-576 |
| Phosphatidylinositol signaling system | 0.028873667 | 12 | miR-425, miR-576 |
| Endocytosis | 0.029179408 | 21 | miR-425, miR-576 |
| Vibrio cholerae infection | 0.029179408 | 10 | miR-425, miR-576 |
| Hepatitis B | 0.029179408 | 18 | miR-425, miR-576 |
| Proteoglycans in cancer | 0.029179408 | 23 | miR-425, miR-576 |
| Regulation of actin cytoskeleton | 0.030390889 | 22 | miR-425, miR-576 |
| TGF-beta signaling pathway | 0.033842977 | 10 | miR-425, miR-576 |
| Bacterial invasion of epithelial cells | 0.039130785 | 10 | miR-425, miR-576 |
| Bladder cancer | 0.039391842 | 8 | miR-425, miR-576 |

**Table S5. Correlation analysis between miR-425/miR-576 and clinical variables in CRC patients (n=157)**

| **Characteristics** | **miR-425** | | **miR-576** | |
| --- | --- | --- | --- | --- |
|  | **Low expression** | **High**  **expression** | **Low expression** | **High**  **expression** |
| **T stage** | | | | |
| T_1_+T_2_ | 25(86.2) | 4(13.8) | 24(82.8) | 5(17.2) |
| T_3_+T_4_ | 67(52.3) | 61(47.7) | 59(46.1) | 69(53.9) |
| *P ^†‡^* | **0.001^†^** | **0.003^‡^** | **<0.001^†^** | **0.001^‡^** |
| *Adjusted OR(95%CI) ^§^* | 1.000 | 5.453(1.766-16.832) | 1.000 | 6.623(2.265-19.371) |
| **N stage** | | | | |
| No | 39(56.5) | 30(43.5) | 40(58.0) | 29(42.0) |
| Yes | 53(60.2) | 35(39.8) | 43(48.9) | 45(51.1) |
| *P ^†‡^* | 0.744**^†^** | 0.809**^‡^** | 0.265**^†^** | 0.267**^‡^** |
| *Adjusted OR(95%CI) ^§^* | 1.000 | 0.922(0.477-1.783) | 1.000 | 1.447(0.754-2.776) |
| **Sample size (cm)** | | | | |
| ≤6 | 45(52.3) | 41(47.7) | 49(57.0) | 37(43.0) |
| >6 | 47(66.2) | 24(33.8) | 34(47.9) | 37(52.1) |
| *P ^†‡^* | 0.103^†^ | 0.065^‡^ | 0.266^†^ | 0.263^‡^ |
| *Adjusted OR(95%CI) ^§^* | 1.000 | 0.536 (0.276-1.040) | 1.000 | 1.441(0.760-2.732) |
| **Intestinal tract occupation** | | | | |
| ≤1/2 | 34(75.6) | 11(24.4) | 36(80.0) | 9(20.0) |
| >1/2 | 58(51.8) | 54(48.2) | 47(42.0) | 65(58.0) |
| *P ^†‡^* | **0.007^†^** | **0.012^‡^** | **<0.001^†^** | **<0.001^‡^** |
| *Adjusted OR(95%CI) ^§^* | 1.000 | 2.745 (1.246-6.048) | 1.000 | 5.226(2.277-11.995) |
| **Differentiation degree** | | | | |
| Grade 1 | 65(76.5) | 20(23.5) | 51(60.0) | 34(40.0) |
| Grade 2 | 27(37.5) | 45(62.5) | 32(44.4) | 40(55.6) |
| *P ^†‡^* | **<0.001^†^** | **<0.001^‡^** | 0.056^†^ | 0.074^‡^ |
| *Adjusted OR(95%CI) ^§^* | 1.000 | 5.082 (2.520-10.249) | 1.000 | 1.811(0.943-3.475) |
| **Vascular invasion** | | | | |
| No | 83(58.9) | 58(41.1) | 75(53.2) | 66(46.8) |
| Yes | 9(56.3) | 7(43.8) | 8(50.0) | 8(50.0) |
| *P ^†‡^* | 1.000^†^ | 0.951^‡^ | 1.000^†^ | 0.761^‡^ |
| *Adjusted OR(95%CI) ^§^* | 1.000 | 1.034 (0.355-3.010) | 1.000 | 1.177(0.412-3.363) |
| **Primary site** | | | | |
| Colon | 54(72.0) | 21(28.0) | 40(53.3) | 35(46.7) |
| Rectum | 38(46.3) | 44(53.7) | 43(52.4) | 39(47.6) |
| *P ^†‡^* | **<0.001^†^** | **0.001^‡^** | 1.000^†^ | 0.570^‡^ |
| *Adjusted OR(95%CI) ^§^* | 1.000 | 3.427 (1.670-7.035) | 1.000 | 1.212(0.625-2.348) |
| **Liver metastasis** | | | | |
| No | 84(65.6) | 44(34.4) | 81(63.3) | 47(36.7) |
| Yes | 8(27.6) | 21(72.4) | 2(6.9) | 27(93.1) |
| *P ^†‡^* | **<0.001^†^** | **<0.001^‡^** | **<0.001^†^** | **<0.001^‡^** |
| *Adjusted OR(95%CI) ^§^* | 1.000 | 5.204 (2.079-13.026) | 1.000 | 23.836(5.387-105.465) |

**P^†^**-values calculated from two-sided χ2 tests or Fisher's exact test.

**P^‡^**-values, Adjusted OR(95%CI) ^§^ calculated by unconditional logistic regression adjusted for **age, sex, and family history**.

**Table S6. [Stratified](../../../../F:/Program%20Files/Youdao/Dict/8.9.4.0/resultui/html/index.html#/javascript:;) correlation analysis between miR-425/miR-576 and clinical variables in CRC patients (n=157)**

| **Characteristics** | **miR-425** | | **miR-576** | |
| --- | --- | --- | --- | --- |
|  | **Low expression** | **High**  **expression** | **Low expression** | **High**  **expression** |
|  | ***Patients with Low miR-576 expression:*** | | ***Patients with Low miR-425 expression:*** | |
| **T stage** | | | | |
| T_1_+T_2_ | 3(60.0) | 2(40.0) | 2(50.0) | 2(50.0) |
| T_3_+T_4_ | 27(39.1) | 42(60.9) | 19(31.1) | 42(68.9) |
| *P ^†‡^* | 0.390^†^ | 0.234^‡^ | 0.589^†^ | 0.238^‡^ |
| *Adjusted OR(95%CI) ^§^* | 1.000 | 3.486(0.447-26.875) | 1.000 | 4.462(0.372-53.451) |
| **N stage** |  |  |  |  |
| No | 28(70.0) | 12(30.0) | 28(71.8) | 11(28.2) |
| Yes | 34(79.1) | 9(20.9) | 34(64.2) | 19(35.8) |
| *P ^†‡^* | 0.450^†^ | 0.413^‡^ | 0.504^†^ | 0.466^‡^ |
| *Adjusted OR(95%CI) ^§^* | 1.000 | 0.653(0.236-1.810) | 1.000 | 1.409(0.561-3.542) |
| **Sample size (cm)** | | | | |
| ≤6 | 12(32.4) | 25(67.6) | 16(39.0) | 25(61.0) |
| >6 | 18(48.6) | 19(51.4) | 5(20.8) | 19(79.2) |
| *P ^†‡^* | 0.236^†^ | 0.194^‡^ | 0.173^†^ | 0.076^‡^ |
| *Adjusted OR(95%CI) ^§^* | 1.000 | 0.525 (0198-1.389) | 1.000 | 3.019(0.891-10.226) |
| **Intestinal tract occupation** | | | | |
| ≤1/2 | 5(55.6) | 4(44.4) | 7(63.6) | 4(36.4) |
| >1/2 | 25(38.5) | 40(61.5) | 14(25.9) | 40(74.1) |
| *P ^†‡^* | 0.471^†^ | 0.185^‡^ | **0.030^†^** | **0.030^‡^** |
| *Adjusted OR(95%CI) ^§^* | 1.000 | 2.804 (0.611-12.876) | 1.000 | 4.769(1.160-19.610) |
| **Differentiation degree** | | | | |
| Grade 1 | **21(61.8)** | **13(38.2)** | 7(35.0) | 13(65.0) |
| Grade 2 | **9(22.5)** | **31(77.5)** | 14(31.1) | 31(68.9) |
| *P ^†‡^* | **0.001^†^** | **0.003^‡^** | 0.780^†^ | 0.869^‡^ |
| *Adjusted OR(95%CI) ^§^* | 1.000 | 4.928 (1.744-13.929) | 1.000 | 1.100(0.352-3.438) |
| **Vascular invasion** | | | | |
| No | 28(42.4) | 38(57.6) | 20(34.5) | 38(65.5) |
| Yes | 2(25.0) | 6(75.0) | 1(14.3) | 6(85.7) |
| *P ^†‡^* | 0.461^†^ | 0.463^‡^ | 0.413^†^ | 0.269^‡^ |
| *Adjusted OR(95%CI) ^§^* | 1.000 | 1.914 (0.338-10.854) | 1.000 | 3.555(0.347-33.756) |
| **Primary site** | | | | |
| Colon | **19(54.3)** | **16(45.7)** | 5(23.8) | 16(72.2) |
| Rectum | **11(28.2)** | **28(71.8)** | 16(36.4) | 28(63.6) |
| *P ^†‡^* | **0.033^†^** | **0.041^‡^** | 0.400^†^ | 0.419^‡^ |
| *Adjusted OR(95%CI) ^§^* | 1.000 | 2.996 (1.048-8.562) | 1.000 | 0.607(0.181-2.035) |
| **Liver metastasis** | | | | |
| No | 23(48.9) | 24(51.1) | 20(45.5) | 24(54.5) |
| Yes | 7(25.9) | 20(74.1) | 1(4.8) | 20(95.2) |
| *P ^†‡^* | 0.084^†^ | **0.036^‡^** | **0.001^†^** | **0.008^‡^** |
| *Adjusted OR(95%CI) ^§^* | 1.000 | 3.303(1.080-10.103) | 1.000 | 17.653(2.149-145.038) |
|  | ***Patients with High miR-576 expression:*** | | ***Patients with High miR-425 expression:*** | |
| **T satge** | | | | |
| T_1_+T_2_ | 22(91.7) | 2(8.3) | 22(88.0) | 3(12.0) |
| T_3_+T_4_ | 40(67.8) | 19(32.2) | 40(59.7) | 27(40.3) |
| *P ^†‡^* | **0.027^†^** | **0.044^‡^** | **0.012^†^** | **0.011^‡^** |
| *Adjusted OR(95%CI) ^§^* | 1.000 | 5.086(1.044-24.778) | 1.000 | 5.660(1.487-21.552) |
| **N stage** | | | | |
| No | 11(37.9) | 18(62.1) | 12(40.0) | 18(60.0) |
| Yes | 19(42.2) | 26(57.8) | 9(25.7) | 26(74.3) |
| *P ^†‡^* | 0.810**^†^** | 0.820^‡^ | 0.290**^†^** | 0.260^‡^ |
| *Adjusted OR(95%CI) ^§^* | 1.000 | 0.889(0.324-2.442) | 1.000 | 1.858(0.633-5.456) |
| **Sample size (cm)** | | | | |
| ≤6 | 33(67.3) | 16(32.7) | 33(73.3) | 12(26.7) |
| >6 | 29(85.3) | 5(14.7) | 29(61.7) | 18(38.3) |
| *P ^†‡^* | 0.077^†^ | 0.058^‡^ | 0.271^†^ | 0.283^‡^ |
| *Adjusted OR(95%CI) ^§^* | 1.000 | 0.328 (0.104-1.037) | 1.000 | 1.663(0.657-4.205) |
| **Intestinal tract occupation** | | | | |
| ≤1/2 | 29(80.6) | 7(19.4) | 29(85.3) | 5(14.7) |
| >1/2 | 33(70.2) | 14(29.8) | 33(56.9) | 25(43.1) |
| *P ^†‡^* | 0.319^†^ | 0.336^‡^ | **0.006^†^** | **0.007^‡^** |
| *Adjusted OR(95%CI) ^§^* | 1.000 | 1.689 (0.581-4.907) | 1.000 | 4.598(1.518-13.930) |
| **Differentiation degree** | | | | |
| Grade 1 | 44(86.3) | 7(13.7) | 44(67.7) | 21(32.3) |
| Grade 2 | 18(56.3) | 14(43.8) | 18(66.7) | 9(33.3) |
| *P ^†‡^* | **0.004^†^** | **0.004^‡^** | 1.000^†^ | 0.783^‡^ |
| *Adjusted OR(95%CI) ^§^* | 1.000 | 4.905 (1.646-14.623) | 1.000 | 1.151(0.423-3.131) |
| **Vascular invasion** | | | | |
| No | 28(42.4) | 38(57.6) | 55(66.3) | 28(33.7) |
| Yes | 2(25.0) | 6(75.0) | 7(77.8) | 2(22.2) |
| *P ^†‡^* | 0.416^†^ | 0.412^‡^ | 0.713^†^ | 0.450^‡^ |
| *Adjusted OR(95%CI) ^§^* | 1.000 | 0.402 (0.046-3.574) | 1.000 | 0.527(0.100-2.784) |
| **Primary site** | | | | |
| Colon | **19(54.3)** | **16(45.7)** | 35(64.8) | 19(35.2) |
| Rectum | **11(28.2)** | **28(71.8)** | 27(71.1) | 11(28.9) |
| *P ^†‡^* | **0.012^†^** | **0.013^‡^** | 0.653^†^ | 0.833^‡^ |
| *Adjusted OR(95%CI) ^§^* | 1.000 | **4.689(1.378-15.958)** | 1.000 | 0.902(0.345-2.355) |
| **Liver metastasis** | | | | |
| No | 61(75.3) | 20(24.7) | **61(72.6)** | **23(27.4)** |
| Yes | 1(50.0) | 1(50.0) | **1(12.5)** | **7(87.5)** |
| *P ^†‡^* | 0.444^†^ | 0.322^‡^ | **0.001^†^** | **0.008^‡^** |
| *Adjusted OR(95%CI) ^§^* | 1.000 | 4.500 (0.229-88.243) | 1.000 | 18.973(2.180-165.150) |

**P^†^**-values calculated from two-sided χ2 tests or Fisher's exact test.

**P^‡^**-values, Adjusted OR(95%CI) ^§^ calculated by unconditional logistic regression adjusted for **age, sex, and family history**.

**Table S7.** **The prognostic information of included CRC patients (n=157)**

| Patient | miR-425 (high/low) | miR-425 (high/low) | DFS state | DFS(months) | OS state | OS (months) |
| --- | --- | --- | --- | --- | --- | --- |
| 1 | 1 | 1 | 1 | 13 | 1 | 13 |
| 2 | 1 | 0 | 1 | 25 | 1 | 25 |
| 3 | 0 | 0 | 1 | 75 | 1 | 82 |
| 4 | 0 | 0 | 0 | 74 | 0 | 74 |
| 5 | 1 | 1 | 1 | 24 | 1 | 24 |
| 6 | 1 | 1 | 1 | 37 | 1 | 37 |
| 7 | 0 | 0 | 0 | 66 | 0 | 66 |
| 8 | 0 | 1 | 1 | 24 | 1 | 24 |
| 9 | 1 | 0 | 1 | 14 | 1 | 14 |
| 10 | 0 | 1 | 0 | 63 | 0 | 63 |
| 11 | 0 | 1 | 0 | 63 | 0 | 63 |
| 12 | 0 | 0 | 1 | 13 | 1 | 13 |
| 13 | 0 | 0 | 1 | 11 | 1 | 11 |
| 14 | 0 | 0 | 1 | 55 | 0 | 60 |
| 15 | 1 | 1 | 1 | 58 | 0 | 58 |
| 16 | 0 | 0 | 1 | 30 | 1 | 30 |
| 17 | 0 | 0 | 1 | 30 | 1 | 36 |
| 18 | 0 | 0 | 0 | 55 | 0 | 55 |
| 19 | 0 | 0 | 0 | 55 | 0 | 55 |
| 20 | 0 | 0 | 0 | 55 | 0 | 55 |
| 21 | 0 | 1 | 1 | 19 | 1 | 19 |
| 22 | 0 | 1 | 1 | 20 | 0 | 28 |
| 23 | 0 | 0 | 1 | 63 | 1 | 63 |
| 24 | 0 | 0 | 0 | 89 | 0 | 89 |
| 25 | 0 | 1 | 0 | 88 | 0 | 88 |
| 26 | 1 | 0 | 0 | 13 | 0 | 13 |
| 27 | 0 | 0 | 0 | 82 | 0 | 82 |
| 28 | 0 | 0 | 1 | 24 | 1 | 24 |
| 29 | 0 | 1 | 1 | 30 | 1 | 30 |
| 30 | 1 | 1 | 1 | 23 | 1 | 23 |
| 31 | 0 | 1 | 1 | 25 | 1 | 25 |
| 32 | 0 | 1 | 1 | 12 | 1 | 18 |
| 33 | 0 | 0 | 1 | 36 | 1 | 36 |
| 34 | 1 | 1 | 0 | 78 | 0 | 78 |
| 35 | 0 | 0 | 1 | 8 | 1 | 8 |
| 36 | 1 | 1 | 1 | 20 | 1 | 20 |
| 37 | 0 | 1 | 1 | 16 | 1 | 24 |
| 38 | 0 | 0 | 1 | 40 | 1 | 40 |
| 39 | 0 | 0 | 1 | 75 | 1 | 75 |
| 40 | 0 | 1 | 1 | 3 | 1 | 15 |
| 41 | 0 | 0 | 1 | 26 | 1 | 26 |
| 42 | 1 | 1 | 1 | 55 | 1 | 61 |
| 43 | 0 | 0 | 1 | 80 | 1 | 80 |
| 44 | 1 | 1 | 1 | 24 | 1 | 24 |
| 45 | 0 | 1 | 0 | 36 | 0 | 36 |
| 46 | 0 | 1 | 1 | 76 | 1 | 76 |
| 47 | 0 | 0 | 0 | 103 | 0 | 103 |
| 48 | 0 | 0 | 1 | 25 | 1 | 25 |
| 49 | 0 | 0 | 0 | 103 | 0 | 103 |
| 50 | 0 | 0 | 0 | 102 | 0 | 102 |
| 51 | 1 | 0 | 1 | 24 | 1 | 24 |
| 52 | 1 | 0 | 1 | 24 | 1 | 30 |
| 53 | 0 | 1 | 1 | 42 | 1 | 42 |
| 54 | 0 | 0 | 0 | 94 | 0 | 94 |
| 55 | 0 | 0 | 1 | 92 | 0 | 100 |
| 56 | 0 | 0 | 0 | 98 | 0 | 98 |
| 57 | 0 | 1 | 1 | 18 | 1 | 24 |
| 58 | 1 | 1 | 1 | 24 | 1 | 24 |
| 59 | 1 | 1 | 1 | 28 | 1 | 24 |
| 60 | 1 | 1 | 1 | 6 | 1 | 6 |
| 61 | 0 | 0 | 1 | 62 | 1 | 62 |
| 62 | 0 | 0 | 1 | 50 | 1 | 50 |
| 63 | 0 | 0 | 0 | 98 | 0 | 98 |
| 64 | 0 | 1 | 1 | 36 | 1 | 36 |
| 65 | 1 | 1 | 0 | 98 | 0 | 98 |
| 66 | 1 | 0 | 1 | 98 | 1 | 98 |
| 67 | 0 | 1 | 0 | 88 | 0 | 88 |
| 68 | 1 | 0 | 1 | 24 | 1 | 24 |
| 69 | 1 | 1 | 0 | 67 | 0 | 67 |
| 70 | 1 | 1 | 1 | 24 | 1 | 24 |
| 71 | 0 | 0 | 1 | 38 | 1 | 38 |
| 72 | 0 | 0 | 1 | 26 | 1 | 26 |
| 73 | 0 | 0 | 0 | 76 | 0 | 76 |
| 74 | 0 | 1 | 1 | 26 | 1 | 26 |
| 75 | 1 | 0 | 1 | 68 | 0 | 73 |
| 76 | 1 | 1 | 1 | 30 | 1 | 30 |
| 77 | 0 | 0 | 0 | 36 | 0 | 36 |
| 78 | 1 | 1 | 1 | 20 | 1 | 26 |
| 79 | 0 | 0 | 1 | 37 | 1 | 37 |
| 80 | 1 | 1 | 1 | 20 | 1 | 20 |
| 81 | 0 | 1 | 1 | 19 | 1 | 25 |
| 82 | 1 | 1 | 1 | 24 | 1 | 24 |
| 83 | 1 | 1 | 1 | 52 | 1 | 52 |
| 84 | 1 | 1 | 1 | 14 | 1 | 14 |
| 85 | 0 | 0 | 0 | 55 | 0 | 55 |
| 86 | 1 | 0 | 1 | 30 | 1 | 30 |
| 87 | 0 | 1 | 0 | 55 | 0 | 55 |
| 88 | 1 | 1 | 1 | 48 | 0 | 54 |
| 89 | 1 | 1 | 1 | 10 | 1 | 10 |
| 90 | 0 | 0 | 0 | 53 | 0 | 53 |
| 91 | 1 | 1 | 1 | 36 | 1 | 36 |
| 92 | 1 | 0 | 1 | 28 | 1 | 28 |
| 93 | 1 | 1 | 1 | 12 | 1 | 12 |
| 94 | 0 | 0 | 0 | 51 | 0 | 51 |
| 95 | 1 | 0 | 1 | 26 | 1 | 26 |
| 96 | 0 | 0 | 0 | 80 | 0 | 80 |
| 97 | 1 | 1 | 1 | 20 | 1 | 20 |
| 98 | 0 | 0 | 0 | 92 | 0 | 92 |
| 99 | 0 | 1 | 1 | 30 | 1 | 30 |
| 100 | 0 | 0 | 0 | 90 | 0 | 90 |
| 101 | 0 | 0 | 0 | 89 | 0 | 89 |
| 102 | 1 | 1 | 1 | 52 | 1 | 52 |
| 103 | 0 | 0 | 0 | 89 | 0 | 89 |
| 104 | 1 | 1 | 1 | 16 | 1 | 16 |
| 105 | 0 | 1 | 1 | 15 | 1 | 15 |
| 106 | 0 | 0 | 0 | 72 | 0 | 72 |
| 107 | 0 | 1 | 0 | 87 | 0 | 87 |
| 108 | 1 | 1 | 1 | 24 | 1 | 24 |
| 109 | 0 | 0 | 1 | 25 | 1 | 25 |
| 110 | 1 | 1 | 1 | 75 | 1 | 75 |
| 111 | 1 | 1 | 1 | 15 | 1 | 20 |
| 112 | 1 | 0 | 1 | 50 | 1 | 50 |
| 113 | 1 | 1 | 1 | 16 | 1 | 16 |
| 114 | 1 | 1 | 1 | 13 | 1 | 13 |
| 115 | 1 | 0 | 1 | 40 | 1 | 40 |
| 116 | 1 | 0 | 1 | 11 | 1 | 11 |
| 117 | 1 | 1 | 1 | 44 | 1 | 50 |
| 118 | 0 | 1 | 1 | 6 | 1 | 15 |
| 119 | 0 | 1 | 1 | 24 | 1 | 24 |
| 120 | 1 | 1 | 1 | 30 | 1 | 37 |
| 121 | 1 | 0 | 1 | 16 | 1 | 16 |
| 122 | 1 | 1 | 1 | 40 | 1 | 40 |
| 123 | 1 | 1 | 1 | 24 | 1 | 30 |
| 124 | 0 | 1 | 1 | 20 | 1 | 20 |
| 125 | 1 | 1 | 1 | 27 | 1 | 27 |
| 126 | 0 | 0 | 1 | 20 | 1 | 20 |
| 127 | 1 | 0 | 1 | 1 | 1 | 1 |
| 128 | 1 | 1 | 1 | 30 | 1 | 30 |
| 129 | 0 | 0 | 1 | 50 | 1 | 50 |
| 130 | 0 | 0 | 1 | 24 | 1 | 24 |
| 131 | 0 | 0 | 0 | 0 | 0 | 0 |
| 132 | 1 | 0 | 0 | 105 | 0 | 105 |
| 133 | 0 | 0 | 0 | 106 | 0 | 106 |
| 134 | 0 | 0 | 0 | 105 | 0 | 105 |
| 135 | 0 | 0 | 0 | 105 | 0 | 105 |
| 136 | 1 | 1 | 1 | 25 | 1 | 25 |
| 137 | 0 | 1 | 1 | 39 | 1 | 39 |
| 138 | 1 | 1 | 1 | 24 | 1 | 24 |
| 139 | 0 | 0 | 0 | 104 | 0 | 104 |
| 140 | 0 | 0 | 0 | 105 | 0 | 105 |
| 141 | 0 | 0 | 0 | 102 | 0 | 102 |
| 142 | 0 | 1 | 1 | 36 | 1 | 36 |
| 143 | 0 | 0 | 0 | 101 | 0 | 101 |
| 144 | 1 | 1 | 1 | 76 | 1 | 76 |
| 145 | 0 | 0 | 0 | 102 | 0 | 102 |
| 146 | 0 | 0 | 1 | 90 | 0 | 97 |
| 147 | 0 | 0 | 0 | 66 | 0 | 66 |
| 148 | 1 | 1 | 1 | 15 | 1 | 15 |
| 149 | 0 | 0 | 1 | 88 | 0 | 96 |
| 150 | 1 | 0 | 0 | 110 | 0 | 110 |
| 151 | 0 | 1 | 0 | 95 | 0 | 95 |
| 152 | 1 | 0 | 1 | 36 | 1 | 36 |
| 153 | 1 | 1 | 1 | 75 | 1 | 75 |
| 154 | 0 | 0 | 1 | 87 | 0 | 93 |
| 155 | 1 | 0 | 1 | 25 | 1 | 25 |
| 156 | 1 | 0 | 1 | 2 | 1 | 2 |
| 157 | 0 | 1 | 1 | 60 | 0 | 64 |

**Table S8. Multivariate Cox regression analysis of the [correlation](../../../mac/AppData/Local/youdao/dict/Application/8.9.3.0/resultui/html/index.html#/javascript:;) between *SENCR* and survival (DFS and OS) in CRC patients (n=157)**

| **Variables** | **DFS** | | | | | | **OS** | | | |
| --- | --- | --- | --- | --- | --- | --- | --- | --- | --- | --- |
|  | **Total n** | | **Events n (%)** | | **Adjusted HR(95%CI)** | **P** | **Total n** | **Events n (%)** | **Adjusted HR(95%CI)** | **P** |
| ***miR-425* expression** | | | | | | | | | | |
| ***All patients*** | | | | | | | | | | |
| Low | 92 | 50(54.3) | | 1(reference) | | - | 92 | 43(46.7) | 1(reference) | - |
| High | 65 | 59(90.8) | | 2.792(1.882-4.143) | | **<0.001** | 65 | 56(86.2) | 3.002(1.987-4.535) | **<0.001** |
| ***Patients with High miR-576 expression:*** | | | | | | | | | | |
| Low | 30 | 22(73.3) | | 1(reference) | | - | 30 | 20(66.7) | 1(reference) | - |
| High | 44 | 41(93.2) | | 1.437(0.827-2.497) | | 0.199 | 44 | 39(88.6) | 1.621(0.916-2.870) | 0.097 |
| ***Patients with Low miR-576 expression:*** | | | | | | | | | | |
| Low | 62 | 28(45.2) | | 1(reference) | | - | 62 | 23(37.1) | 1(reference) | - |
| High | 21 | 18(85.7) | | **3.208(1.728-5.956)** | | **<0.001** | 21 | 17(81.0) | **3.610(1.897-6.870)** | **<0.001** |
| ***Patients with liver metastasis*** | | | | | | | | | | |
| Low | 8 | 4(50.0) | | 1(reference) | | - | 2 | 4(50.0) | 1(reference) | - |
| High | 21 | 17(81.0) | | 2.088(0.676-6.453) | | 0.201 | 21 | 17(81.0) | 2.130(0.693-6.453) | 0.187 |
| ***Patients without liver metastasis*** | | | | | | | | | | |
| Low | 84 | 46(54.8) | | 1(reference) | | - | 84 | 39(46.4) | 1(reference) | - |
| High | 44 | 42(68.8) | | **3.022(1.946-4.693)** | | **<0.001** | 44 | 39(88.6) | **3.194(2.009-5.076)** | **<0.001** |
| ***miR-576* expression** | | | | | | | | | | |
| ***All patients*** | | | | | | | | | | |
| Low | 83 | 46(55.4) | | 1(reference) | | - | 83 | 40(48.2) | 1(reference) | - |
| High | 74 | 63(85.1) | | 2.462(1.655-3.663) | | **<0.001** | 74 | 59(79.7) | 2.394(1.581-3.624) | **<0.001** |
| ***Patients with High miR-425 expression:*** | | | | | | | | | | |
| Low | 21 | 18(85.7) | | 1(reference) | | - | 21 | 17(81.0) | 1(reference) | - |
| High | 44 | 41(92.3) | | 1.147(0.651-2.021) | | 0.635 | 44 | 39(88.6) | 1.148(0.641-2.057) | 0.642 |
| ***Patients with Low miR-425 expression:*** | | | | | | | | | | |
| Low | 62 | 28(45.2) | | 1(reference) | | - | 62 | 23(37.1) | 1(reference) | - |
| High | 30 | 22(73.3) | | 2.460(1.357-4.460) | | **0.029** | 30 | 20(46.7) | 2.339(1.248-4.386) | **0.008** |
| ***Patients with liver metastasis*** | | | | | | | | | | |
| Low | 2 | 0(0.00) | | 1(reference) | | - | 2 | 0(0.00) | 1(reference) | - |
| High | 27 | 21(77.8) | | **+∞** | | 0.981 | 27 | 21(77.8) | **+∞** | 0.981 |
| ***Patients without liver metastasis*** | | | | | | | | | | |
| Low | 81 | 46(56.8) | | 1(reference) | | - | 81 | 40(49.4) | 1(reference) | - |
| High | 47 | 42(89.4) | | **2.764(1.775-4.304)** | | **<0.001** | 47 | 38(80.9) | **2.515(1.579-4.005)** | **<0.001** |

Abbreviations: 95%CI, 95% confidence interval; DFS, Disease-free survival; OS, Overall survival. **P** values, Adjusted HR (95%CI) were assessed using multivariate Cox regression analysis adjusted for **age, sex, and family history**.

**Table S9. Data of mRNAs cluster in Heatmap analysis**

| **Up-regulated mRNA** | **Fold Change** | **Down-regulated mRNA** | **Fold Change** |
| --- | --- | --- | --- |
| TRIM31 | 1.63 | **PTEN** | 1.63 |
| ASGR2 | 1.57 | SELM | 1.59 |
| STC2 | 1.51 | LARP6 | 1.57 |
| C8G | 1.49 | PODXL | 1.54 |
| AK1 | 1.47 | FOXF1 | 1.51 |
| CYP2D6 | 1.42 | FZD6 | 1.51 |
| TRPM4 | 1.42 | ZNF644 | 1.50 |
| TPPP3 | 1.39 | TTC28 | 1.48 |
| KRT7 | 1.38 | RBMXL1 | 1.48 |
| COLEC11 | 1.35 | SH3KBP1 | 1.48 |
| GAS6-AS1 | 1.23 | IFIT1 | 1.47 |
| APOC1P1 | 1.19 | HERC5 | 1.45 |
| MLXIPL | 1.19 | ELOVL5 | 1.44 |
| ELMO3 | 1.16 | TCEAL3 | 1.43 |
| PDXDC1 | 1.15 | PDZRN3 | 1.43 |
| CRIPAK | 1.15 | MAGED1 | 1.42 |
| TM4SF5 | 1.14 | IFI44 | 1.39 |
| BAIAP2L1 | 1.05 | PKN2 | 1.37 |
| SDR16C5 | 1.05 | KLF6 | 1.36 |
| LPCAT4 | 1.05 | GSPT2 | 1.36 |
